# Application of Intensity Ratios to Disentangle Rotational Spectra of Large Molecular Clusters: Trifluoroethylene with up to 6 CO_2_ Molecules

**DOI:** 10.1021/acs.jpca.5c05251

**Published:** 2025-10-31

**Authors:** Kyle C. Gilbert, Sean A. Peebles, Brooks H. Pate, Rebecca A. Peebles

**Affiliations:** § Department of Chemistry, 10695 California State University, Sacramento, 6000 J Street, Sacramento, California 95819, United States; # Department of Chemistry, 2358 University of Virginia, McCormick Rd., Charlottesville, Virginia 22904, United States

## Abstract

A generalizable method of intensity ratio analysis has facilitated identification and deconvolution of rotational spectra for nine mixed trifluoroethylene (TFE)/CO_2_ clusters, (TFE)_*m*_(CO_2_)_*n*_, within a single spectroscopic data set. Molecular complexes with *m* = 1−2 and *n* = 1−6 have been investigated using chirped-pulse (CP) Fourier-transform microwave (FTMW) spectroscopy. Collection of two spectra with different CO_2_ concentrations allowed determination of intensity ratios of rotational transitions, which were then correlated with cluster size and formula. The wide composition range of observed TFE/CO_2_ clusters provides an abundance of data with which to benchmark computational methods for determining compositions and energy ordering of large mixed clusters. Initial computational structural results for experimentally observed species are also presented.

## Graphical abstract

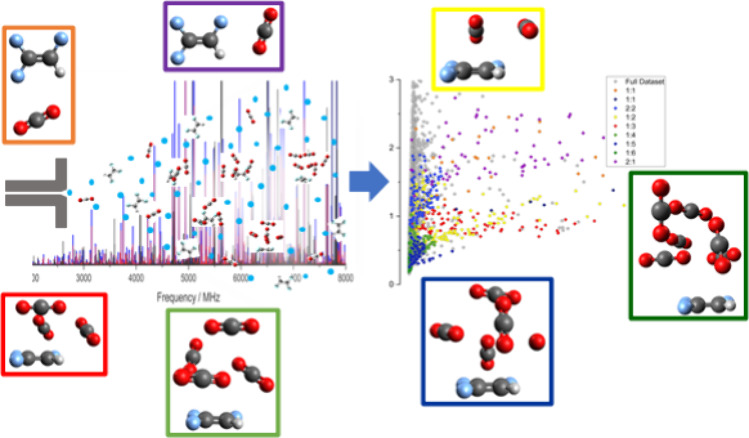

## Introduction

The ability of supercritical carbon dioxide (*sc*-CO_2_) to behave as a solvent toward a wide range of chemical species is of increasing importance as chemists search for less toxic and more environmentally friendly alternatives to “traditional” organic solvents.^1,2^ *sc*-CO_2_ is already used widely for extraction of natural products and as a commercial decaffeination and dry-cleaning agent. Its critical point occurs at relatively mild conditions (*T*_c_ = 31.0 °C and *P*_c_ = 73.8 bar).^3,4^ As a nonpolar molecule with a relatively large molecular quadrupole moment ((−14.31 ± 0.74) × 10^−40^ C m^2^),^5^ CO_2_ is a good solvent for nonpolar and slightly polar species, and it has been shown to be particularly effective for fully fluorinated species, where the solubility of fluorocarbons in *sc*-CO_2_ is significantly higher than the solubility of the equivalent hydrocarbons.^2^

Molecular rotational spectroscopy studies of mixed fluorocarbon/CO_2_ clusters in the gas phase provide important benchmark data for the solvation process through composition-specific characterization of the cluster geometry. These studies face significant theoretical and experimental challenges. For theory, challenges include identifying stable cluster geometries over a range of cluster compositions, obtaining accurate geometries for these weakly bound clusters and calculating accurate relative energies to identify the most stable geometries for each composition. Rotational spectroscopy is uniquely suited to provide experimental data to validate the theoretical methodology. The technique has high sensitivity to cluster geometry, so clusters with different composition, and isomers of clusters with the same composition, can be identified with high confidence. The high spectral resolution of molecular rotational spectroscopy makes it possible to study the complex mixture of cluster composition and isomers produced in a pulsed jet expansion without significant spectral overlap. However, the experimental broadband rotational spectrum is challenging to analyze because of the large number of cluster species present and the wide intensity range across which cluster transitions are observed.

This work presents a general strategy for disentangling these complex spectra with minimal time commitment to data collection. The study investigates mixed clusters of trifluoroethylene (TFE) and CO_2_, with identification of clusters ranging from dimers ((TFE)_1_(CO_2_)_1_) to heptamers ((TFE)_1_(CO_2_)_6_). Two spectra are acquired with different concentration ratios of the fluorocarbon and CO_2_ (plus a spectrum with only fluorocarbon present, so transitions of species that do not contain CO_2_ can be excluded). The ratio of transition intensities in the two measurements is used to filter the data set and extract spectra for clusters of similar composition, building on and extending the extended cross correlation method originally presented by Field.^6,7^ The simplification from the spectral filtering makes it possible to identify individual rotational spectra via pattern recognition, often without requiring an initial guess of candidate geometry from theory. The set of mixed (TFE)_*m*_(CO_2_)_*n*_ clusters identified in the analysis provides benchmarks for testing theoretical methods on large mixed clusters of small molecules, an important area of computational chemistry for which little experimental data exists for comparison.

In earlier work, dimers of CO_2_ with fluoroethylene (FE), 1,1-difluoroethylene (DFE) and TFE were studied,^8–10^ in all cases leading to planar structures with C−H···O interactions that appear to be further stabilized by secondary contacts between the C atom of CO_2_ and one F atom from the fluorinated ethylene. Theoretical calculations for all three 1:1 clusters predicted low-energy nonplanar arrangements with CO_2_ above the C=C bond;^10^ however, these have not been observed experimentally. On the other hand, spectroscopic data on *cis*-1,2-difluoroethylene-CO_2_ definitively show a nonplanar *C*_*s*_ symmetry structure.^11^ More recently, larger mixed FE/CO_2_ and DFE/CO_2_ clusters up to pentamers were observed using microwave spectroscopy.^12,13^ As size increases, it becomes challenging to confirm cluster compositions and to explore potential energy surfaces thoroughly enough to identify the most probable theoretical candidates for cluster structures; however, for mixed FE/CO_2_ and DFE/CO_2_ clusters, one clear conclusion was that molecular arrangements mimic those observed for pure CO_2_ clusters,^14,15^ with FE or DFE behaving more as a “tag” on the outside of a CO_2_ cluster than as a “solute” surrounded by CO_2_ molecules. Recent work from Guo and co-workers on cyclopentene-(CO_2_)_*n*_ clusters (*n* = 1−3) and vinylene carbonate (VC)-(CO_2_)_*n*_ (*n* = 1−5) showed similar results for the arrangements of CO_2_ molecules within each cluster, although there was evidence in the VC-(CO_2_)_4−5_ clusters of CO_2_ beginning to surround a central VC molecule.^16,17^ For TFE, addition of the third fluorine substituent causes the sign of the out-of-plane component of the molecular quadrupole moment to flip from positive, as it is for ethylene, monofluoro- and 1,1- and *cis*-1,2-difluoroethylenes, to negative for TFE.^18^ Because of this key electrostatic difference, it is possible that structural motifs in mixed TFE/CO_2_ clusters could be significantly different than those observed for FE or DFE, and that observations for TFE clusters might more closely mimic the behavior of fully fluorinated species, that typically have high solubilities in *sc*-CO_2_.

A simple data-analytics based method was implemented to facilitate deconvolution of the complex microwave spectra, which had a transition density (with signal-to-noise ratio ≳ 1.5) of ∼1 to 2 lines/MHz, with assignable transitions ranging across 5 orders of magnitude of intensity (strongest dimer lines around 0.2 mV, strongest monomer lines around 30 mV, weakest assignable cluster lines ∼0.2 μV). Our key hypothesis was that transition intensities for clusters with different TFE:CO_2_ ratios would differ from each other in a consistent way when the proportion of CO_2_ in the sample mixture was changed, as was recently reported for FE/CO_2_ clusters.^12^ Variation of parameters other than CO_2_ concentration (e.g., backing pressure, TFE concentration) also would be expected to lead to different intensity ratios for different cluster compositions. For the present work, we selected a constant, low concentration of TFE in comparison to CO_2_ to help minimize self-clustering of TFE molecules, since our primary aim was to observe clusters containing a single TFE. In previously reported FE/CO_2_ cluster studies,^12^ preliminary experiments were performed in which backing pressure was systematically varied, revealing that clusters with different compositions tended to be much less easy to differentiate based on pressure-variation data than with concentration-variation data; thus, pressure-variation was not considered in the present study. It would be of interest to perform a quantitative future investigation of how a wide range of experimental parameters affects intensities of transitions for the full range of observed mixed cluster species; however, for the present work, the complex relationship between transition intensity and sample composition was beyond the scope of our interest. The simple fact that different clusters would have different concentration-intensity relationships was sufficient for our aims.

## Experimental Methods

The chirped-pulse Fourier-transform microwave (CP-FTMW) spectrometer at the University of Virginia (UVa)^19,20^ was used to record three rotational spectra across the 2−8 GHz frequency range. All spectra were recorded in neon carrier gas with 0.2% trifluoroethylene (TFE, Synquest Laboratories). In addition, two of the scans contained 1 and 2% CO_2_, respectively, added to the TFE/Ne mixture (Figure 1a). Spectra were collected using a sample backing pressure of 2.3 atm. For each CO_2_-containing scan, a total of 10^6^ free induction decays (FID) were averaged, with 8 spectra recorded per gas pulse and 3 nozzles operating simultaneously at ambient temperature (∼30 °C). For the TFE-only scan, conditions were identical, and a total of 700,000 FIDs were averaged.

**Figure 1:**
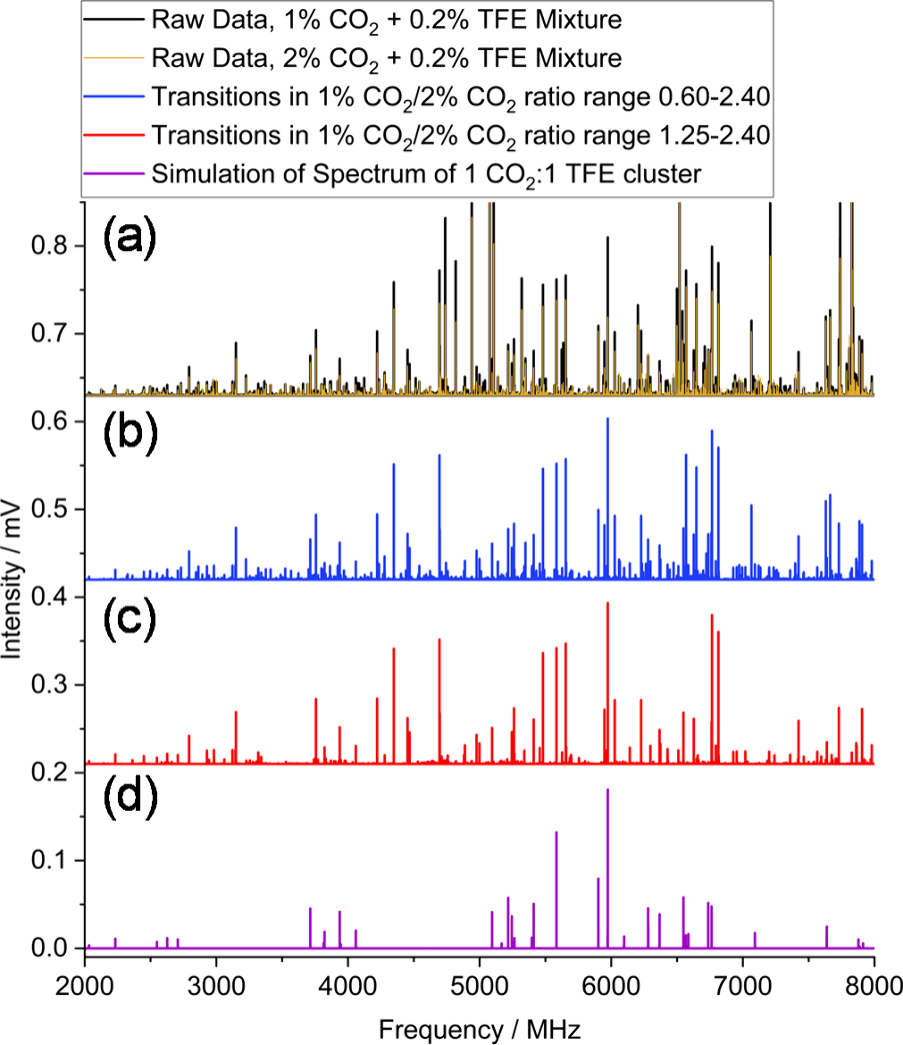
Microwave spectra of trifluoroethylene (TFE)/CO_2_ mixtures (a) with two different sample concentrations of CO_2_ (1% (black) and 2% (gold) with 0.2% TFE); (b, c) showing only transitions from the 1% CO_2_ scan with intensity ratios $\frac{I(1\%\;{\mathrm{CO}}_{2}\;\mathrm{scan})}{I(2\%\;{\mathrm{CO}}_{2}\;\mathrm{scan})}$ in the specified ranges. (d) Simulation of the assigned (TFE)_1_(CO_2_)_1_ cluster spectrum.^10^

Supporting Information (SI) Section I, Figure S1, shows a comparison of the TFE-only scan with scans containing CO_2_.

DFT calculations were performed using Gaussian 16 ^21^ (full Gaussian reference in SI Section II) on the Expanse CPU at the San Diego Super Computer with 32 processors and 4 GB of memory.^22^ Desktop computers running Windows 10 with a minimum of 4 GB of RAM were used for preliminary structural surveys to identify low-energy orientations using ABCluster^23^ with Grimme’s GFN2-xTB method.^24^ These local computers also ran preliminary small basis set optimizations using Gaussian 16W.^21^ Pickett’s SPFIT/SPCAT program^25^ was used in conjunction with Kisiel’s AABS suite^26^ for spectroscopic assignment and fitting. All spectra were fitted using Watson’s *A*-reduction Hamiltonian in the *I* ^r^ representation.^27^

**Figure 2:**
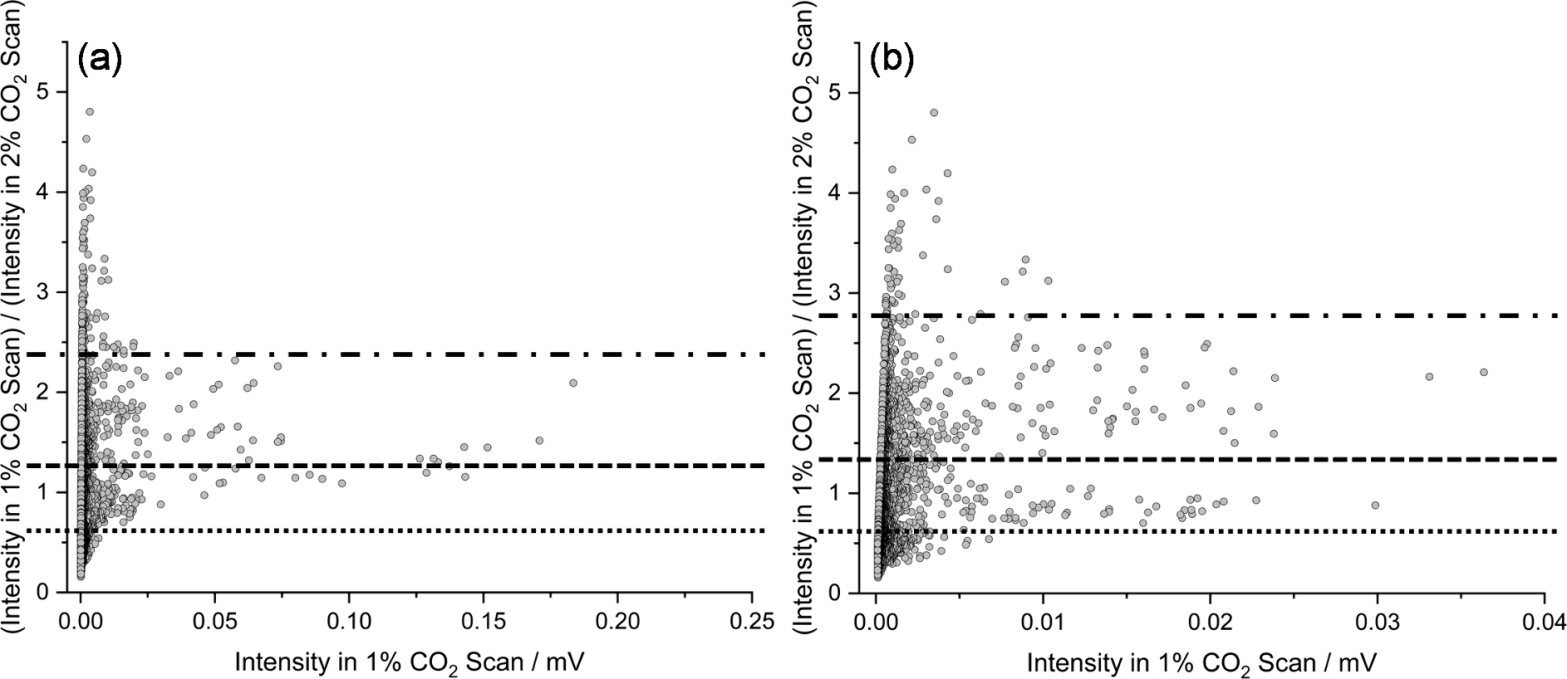
Ratio vs intensity (*RvI*) plots showing $\frac{I(1\%\;{\mathrm{CO}}_{2\;}\mathrm{scan})}{I(2\%\;{\mathrm{CO}}_{2}\;\mathrm{scan})}$ vs intensity in the 1% CO_2_ scan for (a) all transitions that require both TFE and CO_2_ and (b) after subtraction of transitions assigned to the first isomer of the (TFE)_1_(CO_2_)_1_ cluster^10^ and the (TFE)_1_(CO_2_)_2_ cluster. Note the zoomed *x*-axis in (b). Superimposed horizontal lines on both figures delineate ratio ranges used to filter the spectra before further analysis. See text for details.

## Results

The TFE-only scan was used to eliminate transitions not requiring both TFE and CO_2_. Each remaining transition was categorized based on its intensity ratio, $\frac{I(1\%\;{\mathrm{CO}}_{2\;}\mathrm{scan})}{I(2\%\;{\mathrm{CO}}_{2}\;\mathrm{scan})}$ , and its intensity in the 1% CO_2_ scan, leading to a representation of the data which will be referred to as a Ratio vs Intensity or *RvI* plot (Figure 2). The ratio range encompassing the most intense transitions was visually identified, and lines having intensity ratios within that range were isolated and examined independently as a subset of the full data set. A subspectrum isolated in this way typically contained a few hundred transitions (compared to the ∼10,000 transition peak-pick of the raw data), simplifying visual identification of spectroscopic patterns (Figure 1) and therefore greatly facilitating spectroscopic assignment. Once a cluster spectrum was assigned using an isolated subspectrum as a guide, all transitions belonging to that assigned species were removed from the original data set and from the *RvI* plot, allowing the intensity ratio range of the next-strongest unassigned cluster to be identified (Figures 2b and 3). The process was repeated until no clear intensity-spikes across narrow ratio ranges were discernible on the *RvI* plot, indicating that the majority of cluster spectra present had been assigned. This approach is simple, versatile, and easier to implement than an earlier version of the method described in ref 12 (based on Field’s method in refs 6 and 7), which relied on plotting intensities from two spectra against each other for each transition in the spectrum, ideally leading to a graph with points lying along rays, each corresponding to a different cluster species (an Intensity vs Intensity, or *IvI*, plot Figure S2a). That method utilized an additional “density of points” plot to identify boundaries between rays,^12^ which lacked sensitivity to overlapping intensity ratio ranges for different species and which required significant effort to generate compared to the *RvI* plot method implemented here. A strength of the *RvI* plot approach is its simultaneous filtering of transitions based on both intensity ratio and intensity, leading naturally to deconvolution and assignment of spectra with overlapping intensity ratio ranges, as transitions from higher intensity species are removed from the data set, allowing fresh evaluation of the remaining weaker transitions.

**Figure 3:**
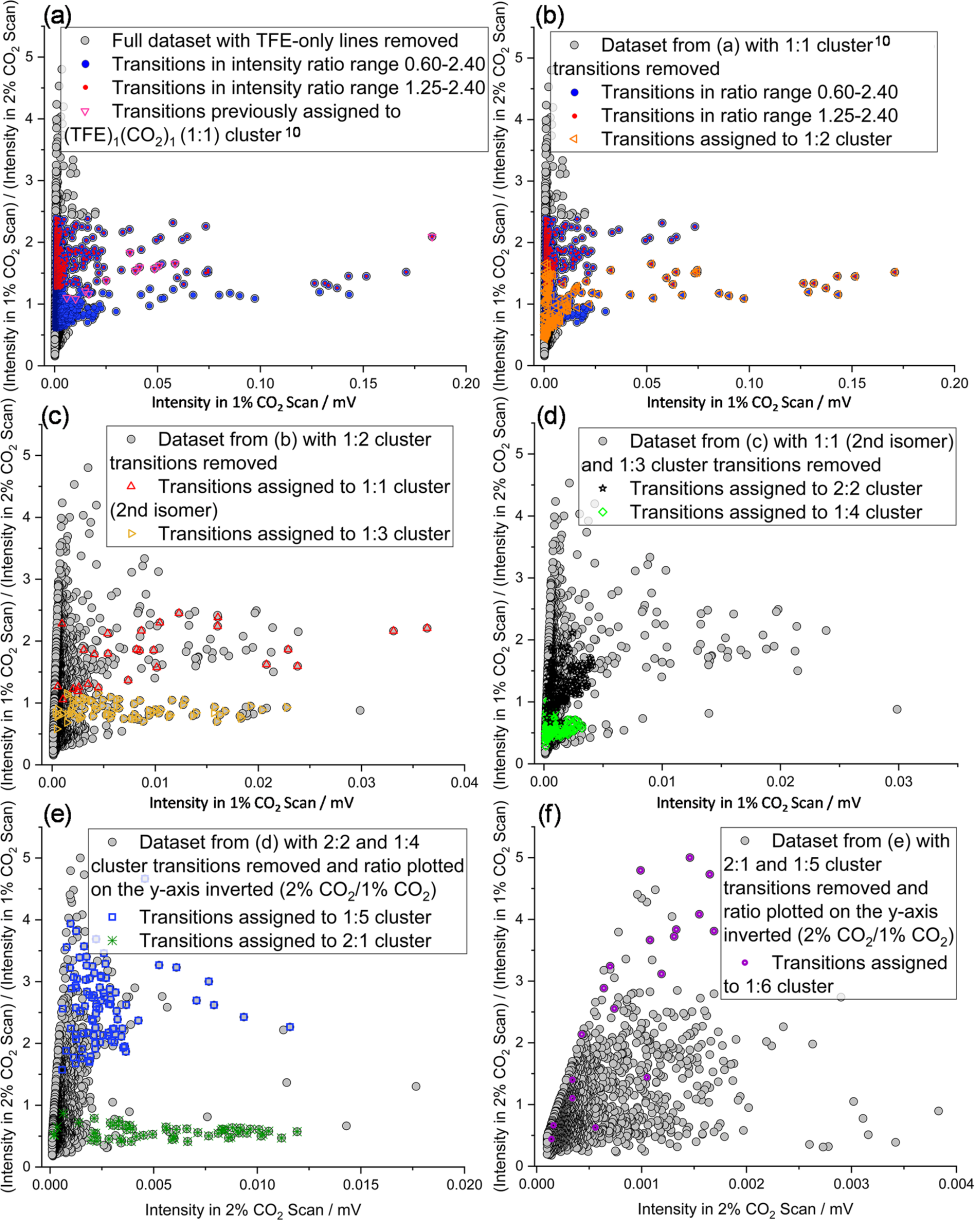
Plots of transition intensity ratio vs transition intensity (*RvI* plots) for samples containing 0.2% TFE and 1 or 2% CO_2_. The ordinate plots the ratio $\frac{I(1\%\;{\mathrm{CO}}_{2}\;\mathrm{scan})}{I(2\%\;{\mathrm{CO}}_{2}\;\mathrm{scan})}$ , unless noted. Colored points for a given species group together. (a) All transitions with signal-to-noise (S/N) ratio over ∼2 in the 1% CO_2_ scan. In (b)−(f), assigned transitions are sequentially removed, revealing new groups of points that may be isolated to help identify spectra of additional molecular clusters (Figure 4). Individual legends describe which transitions have been removed and to which clusters colored transitions correspond.

**Figure 4:**
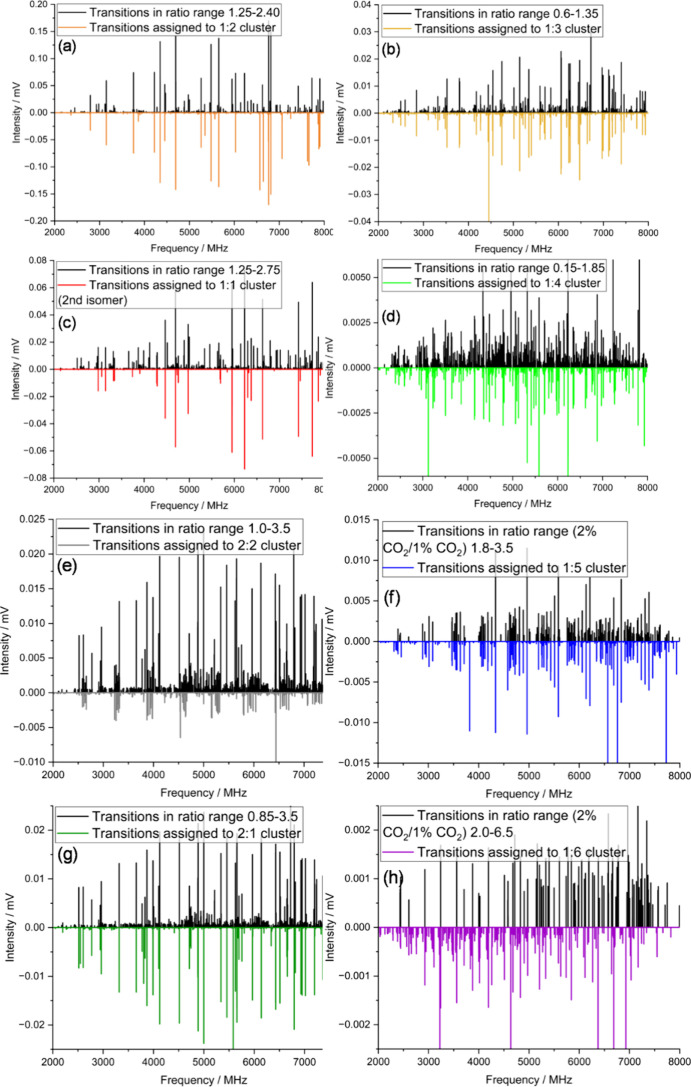
(a)–(h) Spectra displaying transitions within intensity ratio ranges selected using *RvI* plots in Figure 3, allowing identification of patterns attributable to large mixed TFE/CO_2_ clusters. Black traces are subsets of transitions falling within listed ratio ranges from the raw data set; inverted traces are simulations of assigned spectra. Individual legends provide details of intensity ratio ranges (Figure 3) and cluster formulas corresponding to each spectrum.

After removing TFE-only transitions, it was expected that (TFE)_1_(CO_2_)_1_ transitions would dominate the *RvI* plot for the TFE/CO_2_ mixture. This 1:1 cluster provided a proof-of-principle for initial implementation of the *RvI* plot approach, since its microwave spectrum was previously reported.^10^ Examination of Figure 2a shows that most of the intense transitions in the spectrum lie in the ratio range ∼0.60 to 1.25 (between the horizontal dashed and dotted lines in Figure 2a). There is a second block of transitions in the ∼1.25 to 2.40 ratio range (dashed and dash-dot lines in Figure 2a) with about 1/3 of the maximum intensity of the stronger cluster of points. Since this was a new analytical approach for us, and because the groups of points merged smoothly together around ratio ∼1.25 to 1.50, we first examined a spectrum encompassing points in the full 0.60−2.40 ratio range (Figure 1b). Transitions of the previously assigned 1:1 cluster (Figure 1d) were readily apparent in this filtered spectrum; however, to probe whether the 1:1 cluster lies primarily within a narrower part of the 0.60−2.40 range, we also examined a spectrum from the 1.25−2.40 range (Figure 1c). This ratio range was chosen because it encompassed the most intense transitions from the full 0.60−2.40 range, while also largely coinciding with the clear grouping of transitions of slightly lower intensity across the 1.25−2.40 range. Figure 1c shows that this narrower ratio range also would have led to a filtered spectrum that contained the majority of the 1:1 cluster transitions. For this test case, transitions of the relatively abundant 1:1 species are clearly identifiable in the original unfiltered data set (Figure 1a); however, that is not the case for larger, less abundant, clusters.

Following the success of the *RvI* approach in identifying the previously assigned 1:1 cluster,^10^ those assigned transitions were removed from the raw data set and a new *RvI* plot was generated (Figure 3b). Many strong transitions were still present, and the two most intense ratio ranges were essentially unchanged from those that were utilized for the initial analysis. These same ratio ranges were again utilized to generate subspectra containing many fewer transitions than the original data set, with the filtered spectrum for the 1.25−2.40 ratio range shown in Figure 4a. A series of strong, widely spaced doublets dominates this spectrum, and intensities of these transitions are comparable to those of the already assigned 1:1 cluster; however, the doublet pattern is only consistent with the predicted structure and constants of a 1:2 cluster, (TFE)_1_(CO_2_)_2_ (Figure 5, Table 1, SI: Sections III and IV). See below for further details of identification and prediction of cluster formulas and structures.

Analysis continued, following an iterative process of removing assigned transitions, making a new *RvI* plot, isolating a subset of intense transitions across a narrow intensity ratio range, and then using the subspectrum generated from the isolated data set to assign the next cluster in the series. After removal of the assigned transitions for the 1:2 cluster and generation of a new *RvI* plot, the power of intensity ratio based spectrum filtering becomes clear. Figure 2b shows the *RvI* plot obtained when the original 1:1 and the 1:2 cluster transitions are removed. The spectrum containing all transitions on this *RvI* plot is shown in Figure 6a. The *RvI* plot shows two groupings of transitions in the 0.60−1.30 and 1.30−2.75 ratio ranges. Filtered spectra for these two ranges (with slight overlap) are shown in Figures 6b,c. It is clear that the filtration process leads to significant simplification of the spectra, leading to much easier identification and assignment of transitions for additional species than is often possible with more traditional approaches that only involve removal of assigned transitions (similar to the spectrum shown in Figure 6a). Full sets of *RvI* plots and subspectra for all stages of analysis are given in Figures 3 and 4, respectively, and spectroscopic constants for fitted spectra and weighted intensity ratios of assigned transitions for each cluster are reported in Tables 1 and 2. When the strongest remaining transitions had intensity ratios <1, indicating that they were more intense in the 2% CO_2_ scan than the 1% CO_2_ scan, the perspective of analysis was reversed, so that the “parent” spectrum utilized for assignment was the 2% CO_2_ scan, with the *RvI* plot showing $\frac{I(2\%\;{\mathrm{CO}}_{2}\;\mathrm{scan})}{I(1\%\;{\mathrm{CO}}_{2\;}\mathrm{scan})}$ vs Intensity in the 2% CO_2_ scan. That ratio limit was reached at *n* ∼ 4−5 CO_2_ molecules. SI Section VI compares the *RvI* plots with the 1% CO_2_ and 2% CO_2_ parents, highlighting the *n* = 5 transitions (blue squares), which are clearly more easily discernible in the 2% CO_2_ parent plot.

**Figure 5:**
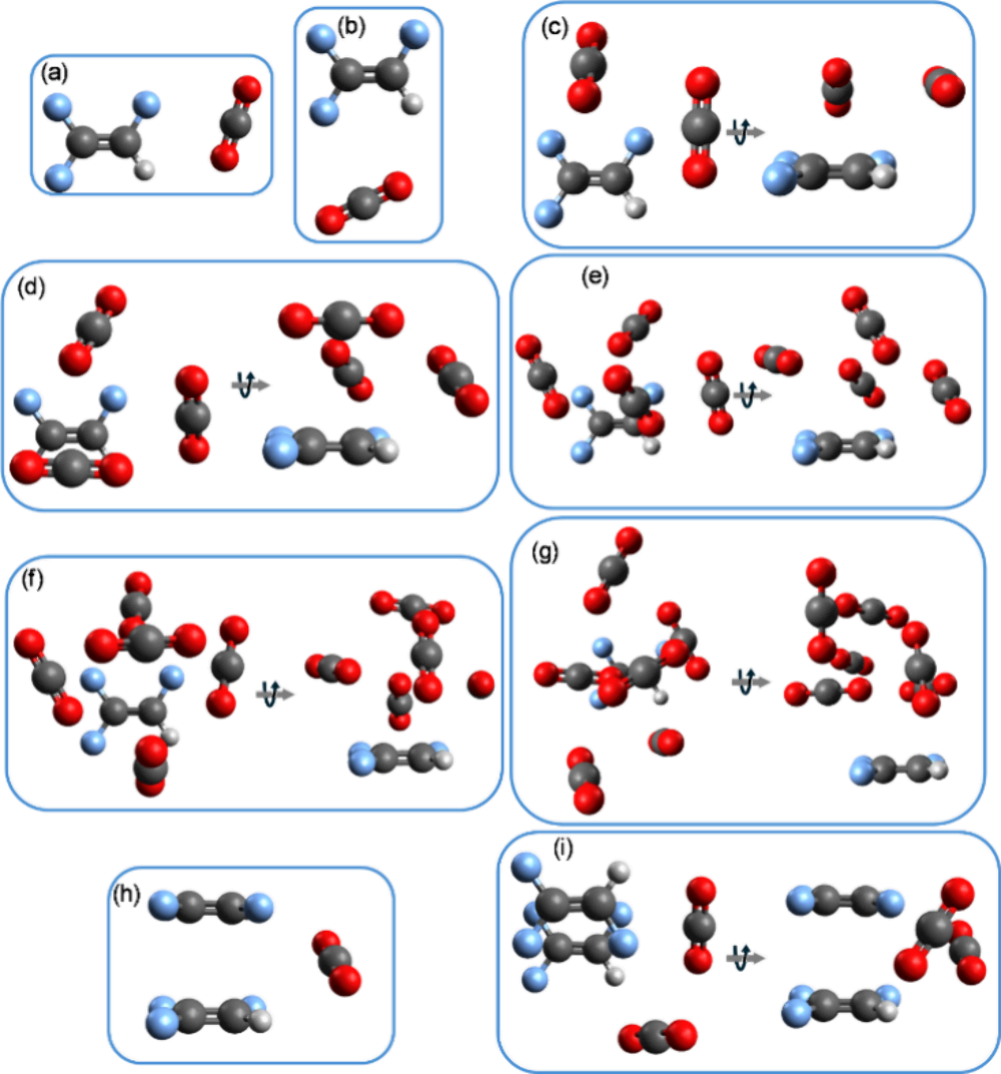
Computed structures of (TFE)_*m*_(CO_2_)_*n*_ clusters with best correspondence between experimental and theoretical rotational constants and dipole moment components. (a) (TFE)_1_(CO_2_)_1_, ref 10; (b) (TFE)_1_(CO_2_)_1_, new isomer; (c) (TFE)_1_(CO_2_)_2_; (d) (TFE)_1_(CO_2_)_3_; (e) (TFE)_1_(CO_2_)_4_; (f) (TFE)_1_(CO_2_)_5_; (g) (TFE)_1_(CO_2_)_6_; (h) (TFE)_2_(CO_2_)_1_; and (i) (TFE)_2_(CO_2_)_2_. See computational details in the SI Section IV and spectroscopic constants in Tables 1 and 2.

The data-centered approach to spectroscopic analysis led to assignment of nine mixed (TFE)_*m*_(CO_2_)_*n*_ clusters, ranging from 2 to 7 molecules, including two isomers of the 1:1 cluster, and two clusters with *m* = 2 TFE molecules (with *n* = 1−2). Tables 1 and 2 summarize the assignments. After removal of all assigned lines from the spectrum, several thousand transitions with S/N ≳ 1.5 remain unassigned. Many of these are likely attributable to as-yet unassigned ^13^C-containing isotopologues for the more intense species and others may indicate the presence of very weak spectra for additional cluster species.

While rotational constants alone provide a reasonable hypothesis as to the size of a cluster whose spectrum has been assigned, the biggest challenge of these studies is confidently identifying the specific formula (the values of *m* and *n* in (TFE)_*m*_(CO_2_)_*n*_) and molecular orientations within a cluster whose spectrum has been fitted. The primary focus of the present work was analysis of clusters containing a single TFE “solute” molecule, but mixed clusters with any TFE:CO_2_ ratio may form, and the *RvI* plot method does not discriminate against clusters with higher numbers of TFE molecules. In the present work, two clusters with *m* = 2 TFE molecules were assigned, and in related ongoing studies of (C_3_F_6_)_*m*_(CO_2_)_*n*_, several pure C_3_F_6_ clusters, C_3_F_6_−Ne_*n*_ clusters, and a (C_3_F_6_)_1_(CO_2_)_1_Ne cluster have been identified using the same methods.^28–30^ Full details of our experimental and computational work toward precise structure determination for observed (TFE)_*m*_(CO_2_)_*n*_ clusters will be presented in a future publication; however, a brief summary of the computational approach used to confirm cluster formulas and to hypothesize potential molecular arrangements for each cluster follows. The *RvI* plot method inherently provides information that assists in identifying compositions of assigned molecular clusters.

For the present series of clusters, an initial series of DFT calculations was performed with ωB97X-D, PBEPBE-D3BJ, and B3LYP-D3BJ functionals and 6−311++G­(2d,2p), aug-cc-pVTZ and aug-cc-pVQZ basis sets using Gaussian 16,^21^ and comparing against experimental constants for two isomers of the 1:1 cluster (Table 1). The choice of functionals and basis sets was driven by a combination of previous experience and reference to the work of Kraus and Frank.^31^ The series of benchmark calculations was also performed for the 1:3 cluster, starting from structures that were determined to be stationary points in preliminary work using rapid CAM-B3LYP/6−31+G­(d,p) calculations. For other (TFE)_1_(CO_2_)_*n*_ clusters with *n* = 1−4, initial orientations were generated based on chemical intuition and structures of previously studied CO_2_-containing clusters.^8–13^ Calculations at the B3LYP-D3BJ/6−311++G­(2d,2p) level ran quickly and gave excellent agreement with experimental rotational constants and with relative magnitudes of dipole moment components, based on observed intensities of *a*-, *b*- and *c*-type transitions (Tables 1 and 2, SI Section IV). Compared to the 6−311++G­(2d,2p) basis set, aug-cc-pVTZ results had similar agreement with experimental constants for 1:1 and 1:3 clusters. Calculations using the quadruple-ζ basis set were prohibitively slow beyond the 1:1 dimer. For further investigation of the potential energy surfaces of the whole series of (TFE)_*m*_(CO_2_)_*n*_ complexes, we opted to employ B3LYP-D3BJ/6−311++G­(2d,2p) calculations. These were implemented in conjunction with initial surveys of potential molecular arrangements from the AI-driven semiempirical model of the ABCluster program, coupled with Grimme’s GFN2-xTB method.^24^

**Figure 6:**
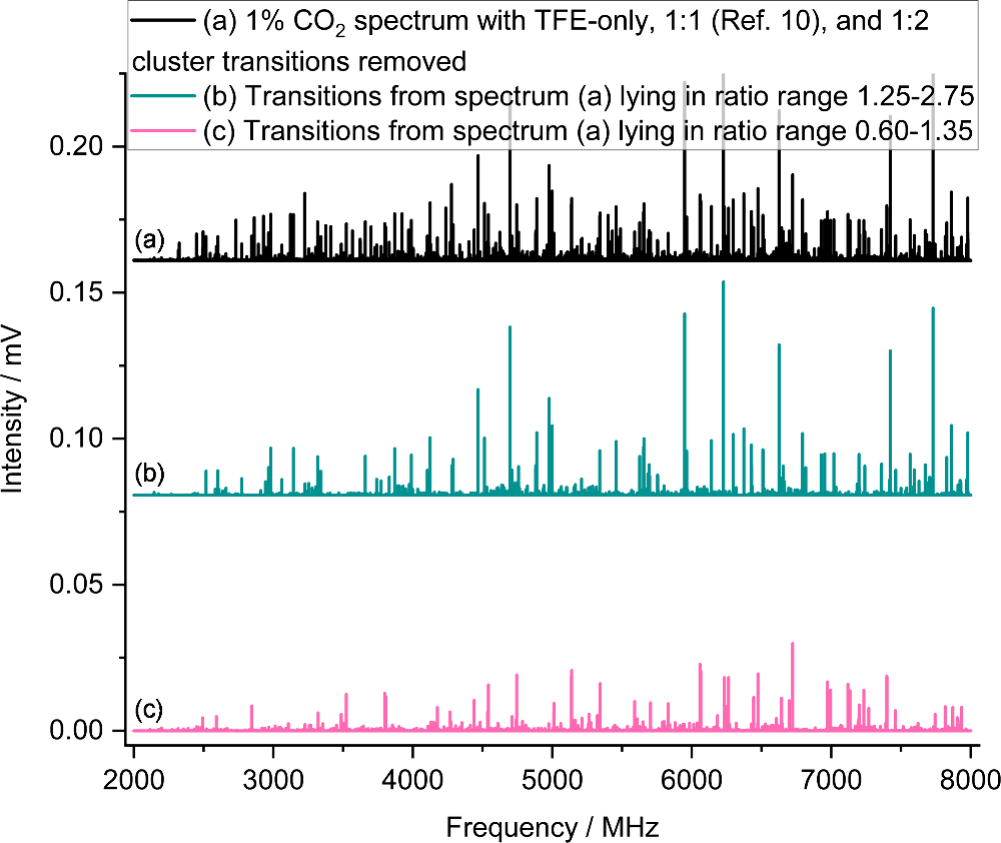
(a) 1% CO_2_ spectrum with transitions requiring only TFE and transitions of the original 1:1 cluster^10^ and the 1:2 cluster removed. (b) Data set from (a) filtered to show only transitions with an intensity ratio $\frac{I(1\%\;{\mathrm{CO}}_{2}\;\mathrm{scan})}{I(2\%\;{\mathrm{CO}}_{2}\;\mathrm{scan})}$ in the 1.25−2.75 range, equivalent to the points between the dashed and dash-dot lines in Figure 2b. (c) Data set from (a) filtered to show only transitions with an intensity ratio $\frac{I(1\%\;{\mathrm{CO}}_{2}\;\mathrm{scan})}{I(2\%\;{\mathrm{CO}}_{2}\;\mathrm{scan})}$ in the 0.60−1.35 range, equivalent to the points between the dotted and dashed lines in Figure 2b. Both filtered spectra are significantly simplified compared to the unfiltered data set in (a), facilitating rapid identification and assignment of spectra.

**Table 1:** Spectroscopic Details and Observed Transition Intensity Ratio Ranges for (TFE)_*m*_(CO_2_)_*n*_ Clusters with *n* + *m* ≤ 3^a^

	(TFE)_1_-(CO_2_)_1_ (ref 10)	(TFE)_1_-(CO_2_)_1_	(TFE)_1_-(CO_2_)_2_	(TFE)_2_ (CO_2_)_1_
*A*/MHz	5355.7872(15)	3585.8666(4)	1233.86103(3)	805.2998(3)
*B*/MHz	696.4235(2)	872.8481(5)	708.593914(16)	538.58382(4)
*C*/MHz	617.32466(15)	702.9889(5)	519.811852(16)	385.88267(3)
Δ_*J*_/kHz	0.4103(15)	0.895(4)	0.42435(14)	0.1252(3)
Δ_*JK*_/kHz	−2.378(10)	−0.76(2)	0.7402(2)	0.3465(15)
Δ_*K*_/kHz	30.7(3)	0^b^	1.5560(5)	0.770(14)
δ_*J*_ */*kHz	49.5(8)	0.1901(7)	0.08080(3)	0.01635(18)
δ_*K*_ */*kHz	0^b^	2.3(2)	0.8016(3)	0.2016(13)
*P*_*aa*_/u Å^2^ ^c^	726.5223(2)	578.4820(4)	637.92842(3)	810.2259(2)
*P*_*bb*_/u Å^2^	92.1377(2)	140.4184(4)	334.30606(3)	499.4442(2)
*P*_*cc*_/u Å^2^	−0.8446(2)	0.5180(4)	75.28545(3)	128.1220(2)
RMS/kHz^d^	2.8	0.9	0.8	0.6
*N* ^d^	45	36	199	65
*A*_theory_/MHz^e^	5320.9 (−0.7%)	3544.8 (−1.1%)	1230.3 (−0.3%)	818.1 (1.6%)
*B*_theory_/MHz	696.8 (0.1%)	880.1 (0.8%)	717.0 (1.2%)	536.2 (−0.4%)
*C*_theory_/MHz	616.1 (−0.2%)	705.0 (0.3%)	526.3 (1.2%)	392.5 (1.7%)
*P*_*aa*, theory_/u Å^2^	725.3	574. 3	627.2	806.2
*P*_*bb*, theory_/u Å^2^	95.0	142.6	333.1	481.4
*P*_*cc*, theory_/u Å^2^	0.0	0.0	77.7	136.3
μ_*a*, theory_/D^f^	0.4 (0.25(3))	1.2 (1.4)	0.2 (0.15)	1.1 (1.0)
μ_*b*, theory_/D	1.2 (1.23(11))	0.7 (0.6)	1.3 (1.0)	0.0 (0.0)
μ_*c*, theory_/D	0.0 (0.0)	0.0 (0.0)	0.2 (0.1)	0.0 (0.0)
μ_*total*, theory_/D	1.2	1.4	1.4	1.1
*m*:*n^g^*	1:1	1:1	1:2	2:1
weighted *R* ^h^	1.74	2.06	1.25	2.00

a SI Section III, Tables S1−S8 list all fitted transitions. SI Section IV reports computational results for each cluster. All reported computational results are from optimizations at B3LYP-B3DJ/6−311++G­(2d,2p) level of theory.

b Undetermined centrifugal distortion constants fixed to a value of zero.

c $P_{aa}=\underset{i}{\sum }m_{i}a_{i}^{2}=\frac{1}{2}\left(I_{b}+I_{c}-I_{a}\right)$ , with permutations for *P*_*bb*_ and *P*_*cc*_.

d RMS=[(∑(νobs−νcalc)2)N]1/2 , where *N* is the number of transitions in the fit, and ν_obs_ and ν_calc_ are observed and calculated transition frequencies, respectively, as reported in Tables S1−S8.

e Computed rotational constants (SI Section IV), with percent difference between computational and experimental values given in parentheses.

f Values in parentheses are estimated relative dipole moment components based on observed experimental intensities, for comparison with theoretical values (first data column is experimental values from ref 10).

g Ratio of # of TFE molecules (*m*) to # of CO_2_ molecules (*n*) in each assigned cluster.

h Weighted $R=\frac{\sum R_{i}I_{i}}{\sum I_{i}}$ , where summations are over all fitted transitions for each cluster, *R*_*i*_ is intensity ratio of 1% CO_2_ scan/2% CO_2_ scan, and *I*_*i*_ is intensity in the 1% CO_2_ scan. Further details in the SI Section V.

**Table 2:** Spectroscopic Details and Observed Transition Intensity Ratio Ranges for (TFE)_*m*_(CO_2_)_*n*_ Clusters with *n* + *m* ≥ 4^a^

	(TFE)_2_-(CO_2_)_2_	(TFE)_1_-(CO_2_)_3_	(TFE)_1_-(CO_2_)_4_	(TFE)_1_-(CO_2_)_5_	(TFE)_1_-(CO_2_)_6_
*A*/MHz	519.09658(8)	667.97830(6)	454.33572(6)	312.42647(5)	264.31076(8)
*B*/MHz	337.73369(3)	483.20615(3)	342.07418(3)	282.23838(3)	209.61718(8)
*C*/MHz	318.51207(4)	439.09806(4)	303.88763(3)	265.61419(5)	158.45311(7)
Δ*_J_*/kHz	0.11943(18)	0.1612(3)	0.05526(15)	0.03110(13)	0.0167(3)
Δ*_JK_*/kHz	−0.1108(9)	0.1869(16)	0.1084(10)	0.0094(6)	0.0128(7)
Δ*_K_*/kHz	0.279(2)	0.119(3)	0.019(2)	0.0064(5)	0^b^
δ*_J_*/kHz	−0.00879(10)	0.03314(15)	0.00279(9)	0.0111(6)	0.00279(13)
δ*_K_*/kHz	0.269(5)	−0.188(3)	0^b^	0^b^	0^b^
*P*_*aa*_/u Å^2^ ^c^	1054.7481(2)	720.12746(12)	1014.0473(2)	1037.8489(4)	1844.1764(15)
*P*_*bb*_/u Å^2^	531.9391(2)	430.82048(12)	648.9984(2)	864.8318(4)	1345.2783(15)
*P*_*cc*_/u Å^2^	441.6351(2)	325.75949(12)	463.3487(2)	752.7616(4)	566.7854(15)
RMS/kHz^d^	1.5	0.8	1.3	1.3	2.2
*N* ^d^	126	85	153	109	77
*A*_theory_/MHz^e^	525.9 (1.3%)	671.5 (0.5%)	459.9 (1.2%)	316.9 (1.4%)	251.2 (−5.0%)
*B*_theory_/MHz	339.6 (0.6%)	489.4 (1.3%)	346.3 (1.2%)	284.0 (0.6%)	209.1 (−0.2%)
*C*_theory_/MHz	320.4 (0.6%)	440.2 (0.3%)	310.8 (2.3%)	270.1 (1.7%)	163.1 (2.9%)
*P*_*aa*, theory_/u Å^2^	1052.3	714.1	993.3	1027.9	1751.8
*P*_*bb*, theory_/u Å^2^	525.1	434.0	632.8	843.2	1346.8
*P*_*cc*, theory_/u Å^2^	435.9	318.5	466.1	751.6	665.1
μ_*a*, theory_/D^f^	0.9 (1.0)	0.2 (0.1)	0.5 (0.3)	0.0 (0.0)	0.5 (0.4)
μ_*b*, theory_/D	0.3 (0.4)	1.1 (1.0)	0.4 (0.3)	0.3 (0.3)	0.9 (1.0)
μ_*c*, theory_/D	0.3 (0.5)	0.5 (0.45)	1.1 (1.0)	1.3 (1.0)	0.6 (0.0)
μ_total, theory_/D	1.0	1.2	1.3	1.3	1.2
*m*:*n* ^g^	2:2	1:3	1:4	1:5	1:6
weighted *R* ^h^	1.34	0.88	0.58	0.45*	0.34*

a SI Section III, Tables S1−S8 list all fitted transitions. SI Section IV reports computational results for each cluster. All reported computational results are from optimizations at B3LYP-B3DJ/6−311++G­(2d,2p) level of theory.

b Undetermined centrifugal distortion constants fixed to a value of zero.

c $P_{aa}=\underset{i}{\sum }m_{i}a_{i}^{2}=\frac{1}{2}\left(I_{b}+I_{c}-I_{a}\right)$ , with permutations for *P*_*bb*_ and *P*_*cc*_.

d RMS=[(∑(νobs−νcalc)2)N]1/2 , where *N* is the number of transitions in the fit, and ν_obs_ and ν_calc_ are observed and calculated transition frequencies, respectively, as reported in Tables S1−S8.

e Computed rotational constants (SI Section IV), with percent difference between computational and experimental values given in parentheses.

f Values in parentheses are estimated relative dipole moment components based on observed experimental intensities, for comparison with theoretical values.

g Ratio of # of TFE molecules (*m*) to # of CO_2_ molecules (*n*) in each assigned cluster.

h Weighted $R=\frac{\sum R_{i}I_{i}}{\sum I_{i}}$ , where summations are over all fitted transitions for each cluster, *R*_i_ is intensity ratio of 1% CO_2_ scan/2% CO_2_ scan, and *I*_i_ is intensity in the 1% CO_2_ scan. Further details in SI Section V. Entries marked * were analyzed using the 2% CO_2_ scan as parent.

ABCluster utilizes the artificial bee colony algorithm to rapidly locate conformations of molecules or clusters; details of this approach have been described elsewhere and will not be discussed in depth here.^23,32–34^ Briefly, for each cluster, an initial run of 2500 randomly generated structures in ABCluster led to a set of very approximate molecular arrangements. Structures were sorted energetically, and rotational constants from the raw output were plotted against energy. The plots showed clear steps in rotational constant values between groups of points belonging to different cluster isomers, allowing facile identification of rotational constant ranges for several most likely structures of each cluster (based on ABCluster’s GFN2 calculations). At the same time, for each cluster, approximately ten lowest energy unique structures from ABCluster were further optimized at B3LYP-D3BJ/6−311++G­(2d,2p) level. Initial benchmarking of this approach on the two observed isomers of the 1:1 cluster indicated that rotational constants predicted using ABCluster in this way are accurate enough to uniquely identify the carrier cluster’s formula for an assigned spectrum (i.e., the differences in constants between different cluster formulas are usually larger than differences between ABCluster’s results and experimental data). For the present study, ABCluster calculations were used to add additional starting orientations for optimizations of clusters up to *n* = 4, and for *n* = 5−6 all DFT optimizations utilized ABCluster results as starting orientations. Formulas for all clusters in this article were also confirmed using ABCluster, where, for example, most clusters with *m* ≠ 1 could be eliminated based on lack of similarity of observed rotational constants to any ABCluster output for those species.

For each experimentally observed cluster (2:1 to 1:6), one computational structure (B3LYP-D3BJ/6−311++G­(2d,2p)) was found to have exceptional agreement with experimentally determined rotational constants and intensities. Favored structures are shown in Figure 5, with computational details in SI Section IV. The aim of these computations was to further confirm cluster formulas and to identify feasible structural arrangements, rather than to perform exhaustive computational structural studies. Emphasis was not put on relative energies of optimized structures, and in some cases, the reported “best structure” here is the second or third lowest energy conformation from the DFT studies (without zero-point energy or basis set superposition error corrections, which could change the energy ordering). It is also possible that transitions of computed lower-energy isomers may account for some of the remaining unassigned lines in the experimental data. In addition, relative energies and energy ordering of different structures varied widely between computational levels and basis sets (while predicted spectroscopic constants, the focus of the present study, were much more consistent). The utility of computational results used in combination with spectroscopic intensity ratios for confirmation of cluster formulas is evident from the example of (TFE)_2_(CO_2_)_2_. This cluster had transitions with overall intensity in the range of observed 1:4 and 1:5 clusters, while the intensity ratio was similar to (TFE)_1_(CO_2_)_1_ clusters. Rotational constants were in the range of the 1:3 to 1:4 clusters. Taken together, this data led to modeling of (TFE)_2_(CO_2_)_2_ clusters and an optimized structure with rotational constants differing by less than 2.5% from experiment and calculated dipole moment components in similarly good agreement with observed transition intensities. It is very unlikely that a different cluster formula or molecular arrangement would lead to such close agreement with observed experimental results. A more in-depth computational study is planned (including incorporating zero-point energy and basis set superposition error corrections and anharmonic frequency calculations) to further probe the full potential energy surface landscapes of the series of (TFE)_*m*_(CO_2_)_*n*_ clusters.

## Discussion

Although we hypothesized that observed structures for the more highly fluorinated TFE clusters might show arrangements of CO_2_ molecules more consistent with a “solvation shell” than was observed for FE and DFE clusters,^35–38^ that does not appear to be the case, at least up to *n* = 6. Observed clusters for *n* = 2−3 show arrangements of CO_2_ molecules that mimic structures observed in pure CO_2_ complexes^14,15^ with a fluorinated ethylene “tag”, rather than displaying a more solvation shell like arrangement of CO_2_ molecules around TFE. These structures are also very similar to those observed for FE, DFE, vinylene carbonate (VC) and cyclopentene (CP).^8,9,17,16^ For *n* = 4, the pure CO_2_ cluster has not been observed experimentally, and there is significant variation in molecular arrangements of (CO_2_)_4_ fragments across all mixed clusters containing 4 CO_2_ molecules observed to-date. Just before this article was submitted, (FE)_1_(CO_2_)_4_ was assigned, allowing comparison of the *n* = 4 clusters across the series of fluorinated ethylenes. While the computational prediction for (CO_2_)_4_ is a roughly tetrahedral arrangement (Figure 7a),^15^ that motif is only observed in the (DFE)_1_(CO_2_)_4_ cluster (Figure 7c).^13^ In (FE)_1_(CO_2_)_4_, the CO_2_ molecules are arranged symmetrically around the F atom (Figure 7b) with pairs of CO_2_ molecules interacting in skewed T-shaped orientations, while in the TFE cluster, the CO_2_ molecules are arranged above the plane of the TFE molecule roughly perpendicular to the C=C bond, and approximately parallel to each other (Figure 7d). None of the CO_2_ molecules in the TFE cluster is in a position similar to those occupied in the observed 1:1 clusters, while in the FE cluster one of the two observed 1:1 isomers forms the core of the 1:4 structure. Wang’s recent study of vinylene carbonate clusters shows a (CO_2_)_4_ fragment similar to that in the FE cluster,^17^ as does Xei et al.’s study of the *n* = 4 cluster with monoethanolamine.^39,40^ Additional spectroscopic studies are in progress on clusters of the fully fluorinated “solute” C_3_F_6_ with CO_2_, and it is hoped that these may show CO_2_ orientations that more closely mimic a solvation shell. While (C_3_F_6_)_1_(CO_2_)_4_ has not yet been observed, the (C_3_F_6_)_1_(CO_2_)_3_ cluster has a propeller-like arrangement of CO_2_ molecules similar to structures in the observed fluoroethylene series. Similar propeller-like (CO_2_)_3_ arrangements are also observed with CP and VC;^16,17^ thus, it appears that significant differences between CO_2_ arrangements begin to appear at the *n* = 4 cluster.

**Figure 7:**
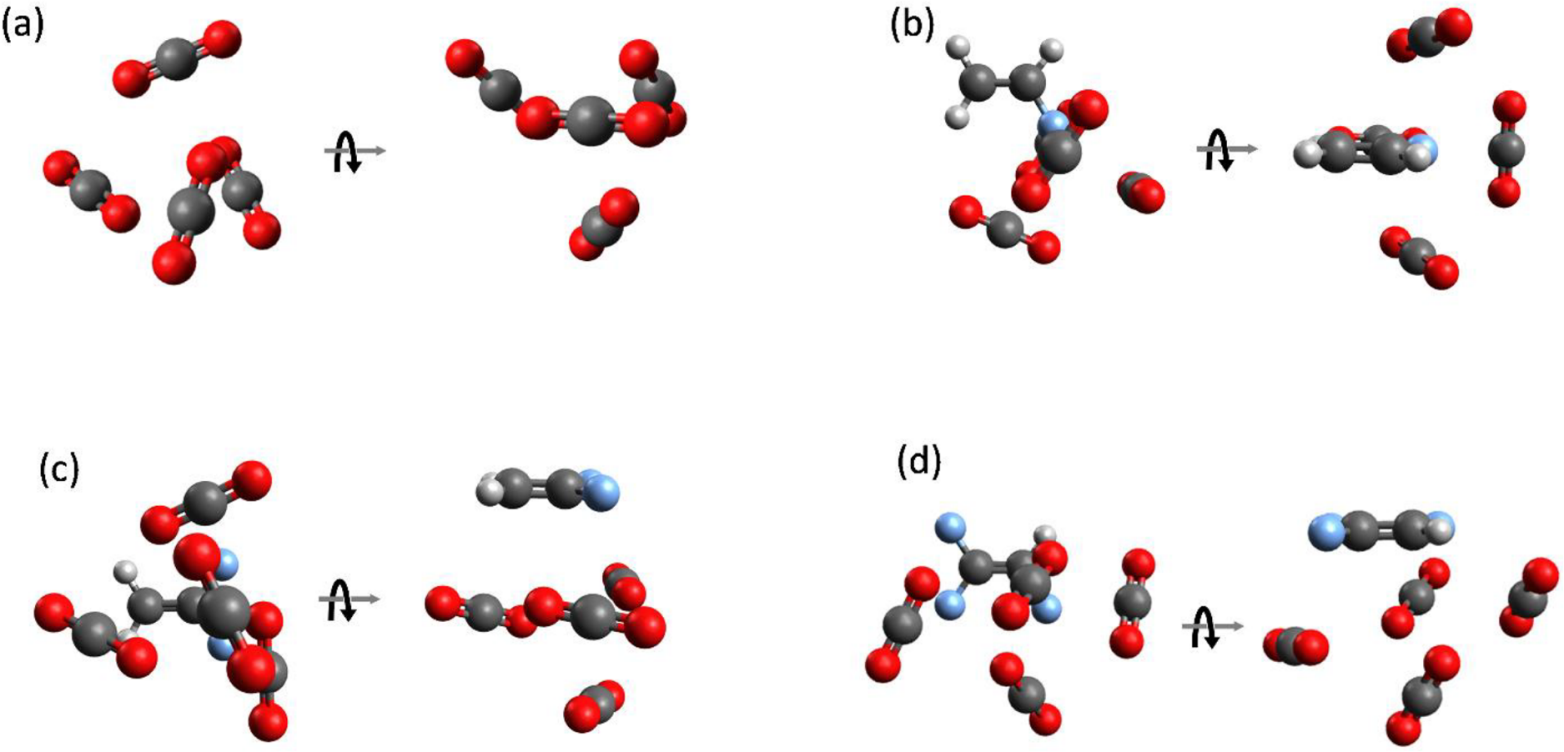
Computed structures for clusters containing 4 CO_2_ molecules. (a) (CO_2_)_4_ from ref 15; (b) (FE)_1_(CO_2_)_4_ (work in progress); (c) (DFE)_1_(CO_2_)_4_ from ref 13; and (d) (TFE)_1_(CO_2_)_4_ from this work.

To our knowledge, (VC)_1_(CO_2_)_5_,^17^ (FE)_1_(CO_2_)_5_ (work in progress), and (TFE)_1_(CO_2_)_5_ are the only 5 CO_2_ clusters to have been observed. All have similar highly symmetric (CO_2_)_5_ fragments, although the “solute” is aligned differently with respect to the CO_2_ molecules in the three species. In the FE and VC clusters, the (CO_2_)_5_ fragment surrounds an electronegative F or O atom, while in the TFE cluster, with the out-of-plane component of the molecular quadrupole moment having opposite sign to that of FE,^18^ the (CO_2_)_5_ fragment is positioned entirely above the TFE plane (Figure 5f). For the TFE-containing clusters, this motif is continued in (TFE)_1_(CO_2_)_6_, where the (CO_2_)_6_ fragment is again completely above the TFE plane (Figure 5g).

## Conclusions

Combining CP-FTMW spectroscopy with a data-centered intensity ratio based analysis method has led to identification of eight new mixed (TFE)_*m*_(CO_2_)_*n*_ clusters and one previously reported (TFE)_1_(CO_2_)_1_ cluster.^10^ A combination of AI-guided semiempirical calculations utilizing ABCluster followed by higher level DFT optimizations was crucial to confirming cluster formulas and identifying likely structural arrangements for each cluster, with B3LYP-D3BJ/6−311++G­(2d,2p) optimizations providing a good balance between computational speed and accuracy (judged by agreement of calculated rotational constants and dipole moment components with experimental constants and observed intensities). Plotting data points for all assigned transitions on *IvI* and *RvI* plots and an analysis of average ratios (Tables 1 and 2, SI Section V) shows clearly that intensity ratio $\left(\frac{I(1\%\;{\mathrm{CO}}_{2\;}\mathrm{scan})}{I(2\%\;{\mathrm{CO}}_{2\;}\mathrm{scan})}\right)$ decreases as the number of CO_2_ molecules in a cluster increases; however, the range of ratios observed for most clusters is wide, with significant overlap between species. Separating species based on the combination of intensity ratios and absolute intensities, while successively removing assigned transitions from the data set, provides a straightforward way to overcome the challenges of overlapping intensity ranges and to deconvolute these dense spectra of complex mixtures of clusters.

Observed (TFE)_1_(CO_2_)_*n*_ clusters up to *n* = 3 have arrangements of CO_2_ molecules that mimic pure CO_2_ clusters. For *n* = 4−6, CO_2_ molecules still do not arrange in ways that mimic what might be expected for a solvation shell surrounding the TFE molecule; however, the arrangements of the (CO_2_)_*n*_ fragments also appear to be significantly different than those observed for pure CO_2_ clusters.

## Supplementary Material



## Notes

Raw spectra, the full refs 17 and 21, tables listing all fitted spectroscopic transitions; computational results for assigned clusters; analysis/overview of observed intensity ratio ranges for all species; and comparison of *RvI* plots with the 1% CO_2_ spectrum and the 2% CO_2_ spectrum taken as the parent species (PDF)
