# Supplementary material for: Application of Intensity Ratios to Disentangle Rotational Spectra of Large Molecular Clusters: Trifluoroethylene with up to 6 CO2 Molecules
Source: J Phys Chem A. 2025 Oct 31;129(45):10393–403. doi: 10.1021/acs.jpca.5c05251 (PMC12621246; doi:10.1021/acs.jpca.5c05251)

Supplementary Information

Application of intensity ratios to disentangle rotational spectra of large molecular clusters: Trifluoroethylene with up to 6 CO_2 molecules

Kyle C. Gilbert[§], Sean A. Peebles[§], Brooks H. Pate[#], Rebecca A. Peebles[§,*]

§ Department of Chemistry, California State University Sacramento, 6000 J Street, Sacramento, CA 95819, USA
# Department of Chemistry, University of Virginia, McCormick Rd., Charlottesville, Virginia 22904, USA

* Corresponding Author: (916)278-4534, r.a.peebles@csus.edu

Table of Contents

I. Raw Spectra

II. Full references 17 and 21

III. Tables of Fitted Transitions for all Observed Clusters

IV. Computational Results for all Observed Clusters

V. *IvI* and *RvI* Plots with Overlays of All Assigned Transitions and Analysis of Observed Ratio Ranges

VI. Comparison of *RvI* Plots Generated with 1% and 2% CO_2 Parent Spectrum

I. Raw Spectra.

Figure S1. All three microwave scans. All scans contain 0.2% trifluoroethylene (TFE) with neon carrier gas. Panel (a) is the TFE-only sample, for comparison with (b) the 1% CO_2 sample and (c) the 2% CO_2 sample.

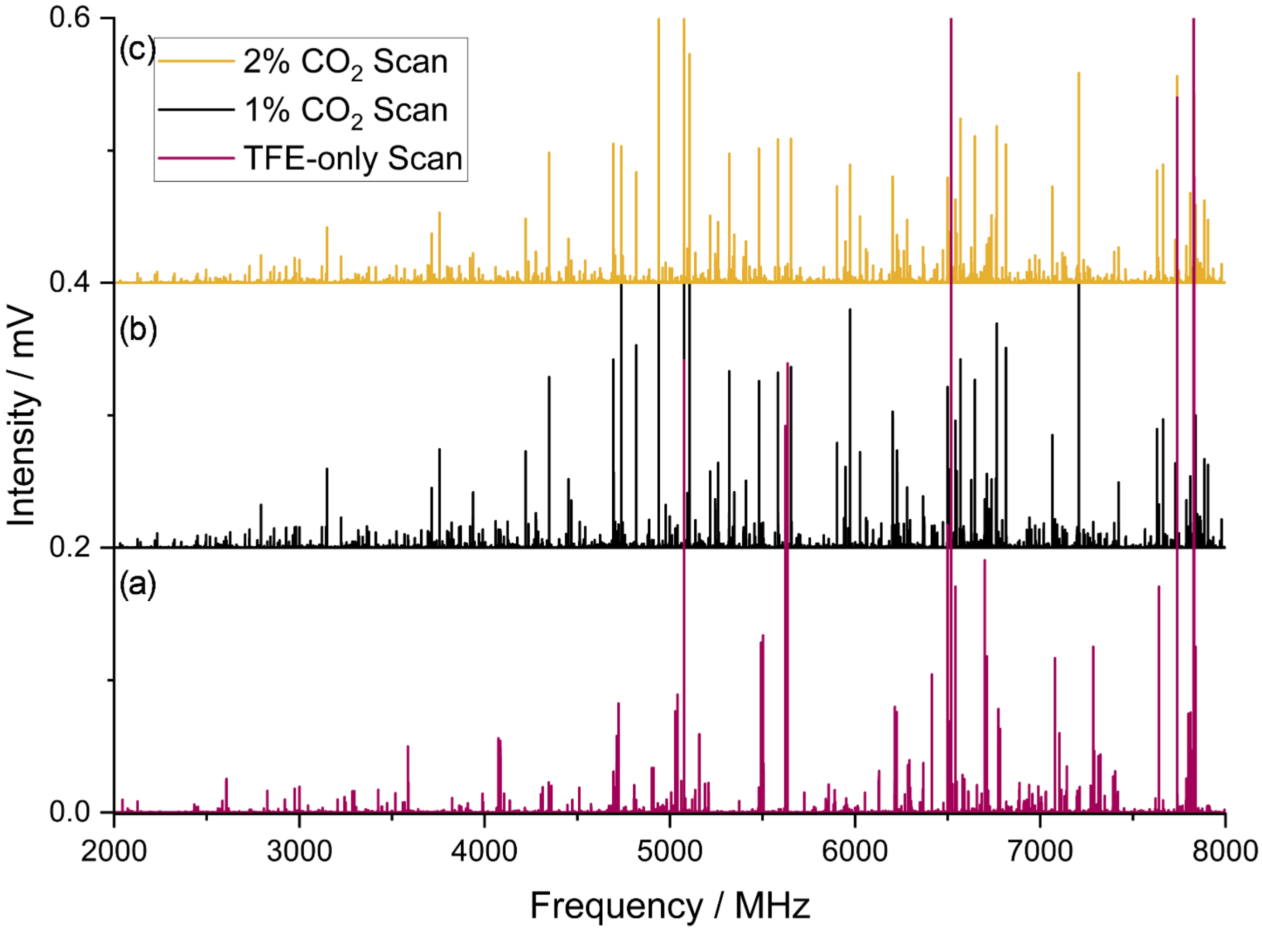

II. Full references 17 and 21:

17. Wang, C.; Lan, J.; Li, M.; Wang, H.; Xie, F.; Tian, X.; Gao, T.; Chen, J.; Yu, Z.; Schnell, M.; Grabow, J.-U.; Gou, Q. Deciphering the Role of Vinylene Carbonate in Shaping CO_2 Cluster Growth and Stability via Wavelet-Enhanced Microwave Spectroscopy, *J. Am. Chem. Soc.*, **2025**, *147*, 4689−4694.

21. Gaussian 16, Revision C.01, M. J. Frisch, G. W. Trucks, H. B. Schlegel, G. E. Scuseria, M. A. Robb, J. R. Cheeseman, G. Scalmani, V. Barone, G. A. Petersson, H. Nakatsuji, X. Li, M. Caricato, A. V. Marenich, J. Bloino, B. G. Janesko, R. Gomperts, B. Mennucci, H. P. Hratchian, J. V. Ortiz, A. F. Izmaylov, J. L. Sonnenberg, D. Williams-Young, F. Ding, F. Lipparini, F. Egidi, J. Goings, B. Peng, A. Petrone, T. Henderson, D. Ranasinghe, V. G. Zakrzewski, J. Gao, N. Rega, G. Zheng, W. Liang, M. Hada, M. Ehara, K. Toyota, R. Fukuda, J. Hasegawa, M. Ishida, T. Nakajima, Y. Honda, O. Kitao, H. Nakai, T. Vreven, K. Throssell, J. A. Montgomery, Jr., J. E. Peralta, F. Ogliaro, M. J. Bearpark, J. J. Heyd, E. N. Brothers, K. N. Kudin, V. N. Staroverov, T. A. Keith, R. Kobayashi, J. Normand, K. Raghavachari, A. P. Rendell, J. C. Burant, S. S. Iyengar, J. Tomasi, M. Cossi, J. M. Millam, M. Klene, C. Adamo, R. Cammi, J. W. Ochterski, R. L. Martin, K. Morokuma, O. Farkas, J. B. Foresman, and D. J. Fox, Gaussian, Inc., Wallingford CT, 2019.

III. Tables of Fitted Transitions for all Observed Clusters. Complete lists of fitted transition frequencies and residuals for all assigned $(TFE)_m(CO_2)_n$ clusters. Results for the more abundant $(TFE)_1(CO_2)_1$ isomer are included in Ref. 10 of the main article.

Table S1. Results for the 2nd $(TFE)_1(CO_2)_1$ isomer.

J'	*K$_a$'*	*K$_c$'*	*J"*	*K$_a$"*	*K$_c$"*	ν / MHz	ν_{obs} - ν_{calc} / MHz
3	0	3	2	1	2	2145.7038	-0.0016
5	1	4	5	1	5	2541.2721	-0.0012
1	1	0	1	0	1	2882.8733	-0.0005
2	1	2	1	1	1	2981.8041	-0.0003
2	1	1	2	0	2	3060.4472	-0.0001
2	0	2	1	0	1	3143.9174	-0.0014
2	1	1	1	1	0	3321.4921	-0.0002
3	1	2	3	0	3	3341.0623	0.0001
6	1	5	6	1	6	3547.8520	0.0014
4	1	3	4	0	4	3740.9005	-0.0007
4	0	4	3	1	3	3904.5763	0.0000
5	1	4	5	0	5	4279.1611	-0.0001
1	1	1	0	0	0	4288.8589	0.0000
3	1	3	2	1	2	4467.9422	-0.0001
3	0	3	2	0	2	4696.6165	0.0000
3	2	2	2	2	1	4727.4332	0.0007
3	2	1	2	2	0	4758.2296	-0.0007
3	1	2	2	1	1	4977.2314	0.0000
5	0	5	4	1	4	5684.7785	0.0030
2	1	2	1	0	1	5694.8314	0.0015
7	1	6	7	0	7	5841.8067	0.0005
4	1	4	3	1	3	5948.6670	0.0001
4	0	4	3	0	3	6226.8128	-0.0002
4	2	3	3	2	2	6297.1364	-0.0001
4	3	2	3	3	1	6317.9821	0.0020
4	3	1	3	3	0	6319.2965	-0.0009
4	2	2	3	2	1	6373.4371	0.0000
4	1	3	3	1	2	6626.6517	-0.0003
8	1	7	8	0	8	6885.0261	-0.0006
3	1	3	2	0	2	7018.8531	-0.0001
5	1	5	4	1	4	7422.6634	0.0000
6	0	6	5	1	5	7462.0874	-0.0014
5	0	5	4	0	4	7728.8658	-0.0001
5	2	4	4	2	3	7861.6204	0.0001
5	3	3	4	3	2	7903.1210	0.0004
5	3	2	4	3	1	7907.7166	-0.0009

Table S2. Results for $(TFE)_1(CO_2)_2$.

J'	*K_a'*	*K_c'*	*J"*	*K_a"*	*K_c"*	ν / MHz	$\nu_{obs} - \nu_{calc}$ / MHz
5	1	5	4	2	2	2137.5543	0.0009
2	2	1	2	1	2	2142.1019	-0.0012
6	2	4	6	1	5	2219.6972	0.0006
2	1	2	1	1	1	2268.0216	0.0028
4	2	2	3	3	1	2293.2838	0.0000
7	3	4	7	2	5	2319.6190	-0.0003
6	3	3	6	2	4	2325.6867	-0.0002
3	1	2	2	2	1	2362.3316	0.0008
2	0	2	1	0	1	2414.3915	0.0000
3	2	2	3	1	3	2449.3345	-0.0009
5	3	2	5	2	3	2496.6852	0.0006
8	3	5	8	2	6	2547.8777	-0.0006
2	1	1	1	1	0	2645.5725	0.0012
5	1	4	5	0	5	2729.7422	-0.0003
4	3	1	4	2	2	2731.3650	-0.0003
5	2	4	4	3	1	2781.2136	-0.0003
2	1	2	1	0	1	2793.2849	0.0001
4	2	3	4	1	4	2862.9958	0.0004
7	2	5	7	1	6	2892.7961	0.0007
3	3	0	3	2	1	2928.7826	-0.0004
9	3	6	9	2	7	3041.8463	0.0010
10	4	6	10	3	7	3048.3479	0.0008
9	4	5	9	3	6	3074.0683	0.0006
3	3	1	3	2	2	3122.2302	-0.0009
3	0	3	2	1	2	3149.0990	-0.0001
6	3	4	5	4	1	3190.0007	0.0013
4	3	2	4	2	3	3225.1182	-0.0007
11	4	7	11	3	8	3296.0210	0.0009
8	4	4	8	3	5	3302.2351	0.0011
5	2	4	5	1	5	3375.0252	-0.0006
3	1	3	2	1	2	3377.9204	-0.0010
5	3	3	5	2	4	3412.1927	0.0000
7	4	3	6	5	2	3427.9363	0.0007
3	0	3	2	0	2	3527.9931	0.0007
6	1	5	6	0	6	3567.1243	-0.0009
6	3	3	5	4	2	3616.0670	0.0001
6	2	5	5	3	2	3617.0416	-0.0001
7	4	3	7	3	4	3623.1941	0.0012
3	2	2	2	2	1	3685.1544	0.0006
6	3	4	6	2	5	3700.4316	0.0003

8	2	6	8	1	7	3728.6399	-0.0005
13	5	8	13	4	9	3751.9325	-0.0005
3	1	3	2	0	2	3756.8141	-0.0005
10	3	7	10	2	8	3781.8088	0.0001
12	5	7	12	4	8	3785.9033	0.0009
12	4	8	12	3	9	3840.9307	-0.0008
3	2	1	2	2	0	3842.3337	0.0008
4	1	3	3	2	2	3863.1717	-0.0003
5	2	3	4	3	2	3894.8062	-0.0001
6	4	2	6	3	3	3921.7378	0.0002
3	1	2	2	1	1	3938.1031	0.0000
6	2	5	6	1	6	3968.9650	-0.0004
11	5	6	11	4	7	4058.0427	0.0005
7	3	5	7	2	6	4096.1353	0.0002
5	4	1	5	3	2	4127.4733	-0.0003
7	2	6	6	3	3	4173.6192	-0.0003
2	2	1	1	1	0	4221.3429	-0.0007
4	4	0	4	3	1	4236.0779	-0.0005
2	2	0	1	1	0	4263.7512	0.0010
9	2	8	8	3	5	4276.6024	-0.0006
4	4	1	4	3	2	4277.0038	-0.0006
5	4	2	5	3	3	4277.4325	0.0003
6	4	3	6	3	4	4310.1415	-0.0004
4	0	4	3	1	3	4348.1317	-0.0003
8	2	7	7	3	4	4393.6101	-0.0001
7	4	4	7	3	5	4402.0207	0.0006
7	1	6	7	0	7	4403.3927	-0.0004
2	2	1	1	1	1	4410.1193	-0.0025
2	2	0	1	1	1	4452.5278	-0.0005
4	1	4	3	1	3	4466.4422	-0.0002
4	0	4	3	0	3	4576.9525	-0.0018
8	4	5	8	3	6	4578.6850	-0.0008
8	3	6	8	2	7	4593.9001	0.0000
8	4	5	7	5	2	4615.3525	-0.0008
7	2	6	7	1	7	4623.5325	0.0010
9	2	7	9	1	8	4630.0806	0.0004
13	4	9	13	3	10	4648.2555	0.0002
11	3	8	11	2	9	4687.9282	0.0004
4	1	4	3	0	3	4695.2648	0.0000
9	5	4	8	6	3	4751.9641	0.0003
9	5	4	9	4	5	4842.2455	0.0026
9	4	6	9	3	7	4858.4276	0.0000

4	2	3	3	2	2	4880.1024	0.0000
3	1	2	2	0	2	4883.3275	0.0003
8	4	4	7	5	3	4913.4728	0.0005
4	3	1	3	3	0	5018.5099	-0.0018
8	5	3	8	4	4	5140.2715	0.0001
9	3	7	9	2	8	5178.3343	0.0000
4	1	3	3	1	2	5185.9954	0.0004
8	1	7	8	0	8	5208.5530	0.0002
4	2	2	3	2	1	5215.9296	0.0002
13	6	7	13	5	8	5234.6889	-0.0009
10	4	7	10	3	8	5248.3248	0.0014
3	2	2	2	1	1	5260.9263	0.0001
7	3	4	6	4	3	5265.7339	0.0000
8	2	7	8	1	8	5317.8058	0.0001
7	5	2	7	4	3	5327.6500	0.0001
5	1	4	4	2	3	5347.9991	0.0000
8	3	6	7	4	3	5364.3177	0.0004
8	5	4	8	4	5	5412.5060	0.0004
7	5	3	7	4	4	5430.3352	0.0002
9	5	5	9	4	6	5430.5037	0.0003
6	5	1	6	4	2	5430.6509	0.0001
3	2	1	2	1	1	5460.5118	0.0001
6	5	2	6	4	3	5460.9911	0.0000
5	0	5	4	1	4	5481.2519	0.0001
5	5	0	5	4	1	5483.2038	0.0001
5	5	1	5	4	2	5489.5907	-0.0006
10	5	6	10	4	7	5511.6207	-0.0002
10	2	8	10	1	9	5518.2780	-0.0002
5	1	5	4	1	4	5535.9619	0.0006
6	2	4	5	3	3	5567.8120	0.0000
5	0	5	4	0	4	5599.5617	-0.0005
12	3	9	12	2	10	5651.8292	0.0005
5	1	5	4	0	4	5654.2714	-0.0003
11	5	7	11	4	8	5681.4415	0.0007
12	6	6	12	5	7	5706.9523	-0.0007
11	4	8	11	3	9	5743.2313	-0.0001
3	2	2	2	1	2	5827.2563	-0.0005
9	4	6	8	5	3	5870.8587	-0.0008
9	1	8	9	0	9	5984.5674	-0.0004
10	5	6	9	6	3	6022.7075	-0.0022
3	2	1	2	1	2	6026.8419	-0.0005
9	2	8	9	1	9	6035.1113	0.0003

5	2	4	4	2	3	6047.9906	-0.0012
11	6	5	11	5	6	6091.8614	0.0004
9	3	7	8	4	4	6152.7024	-0.0009
4	2	3	3	1	2	6202.9249	-0.0003
10	5	5	9	6	4	6216.1034	0.0010
5	4	2	4	4	1	6235.4935	0.0000
4	3	1	4	0	4	6248.0831	-0.0005
12	4	9	12	3	10	6327.7659	-0.0001
13	5	9	13	4	10	6347.8832	0.0004
5	3	2	4	3	1	6349.8316	-0.0002
10	6	4	10	5	5	6356.7236	0.0003
11	2	9	11	1	10	6362.1115	-0.0006
5	1	4	4	1	3	6364.9288	-0.0006
11	6	6	11	5	7	6497.0048	0.0001
12	6	7	12	5	8	6501.1015	0.0002
9	6	3	9	5	4	6521.0817	0.0000
11	3	9	11	2	10	6522.4583	-0.0007
10	6	5	10	5	6	6533.5694	-0.0003
4	1	3	3	0	3	6541.3296	-0.0002
6	0	6	5	1	5	6568.2088	-0.0001
5	2	3	4	2	2	6584.5117	-0.0008
9	6	4	9	5	5	6586.6263	0.0000
6	1	6	5	1	5	6591.7216	0.0014
8	6	2	8	5	3	6619.0648	-0.0010
6	0	6	5	0	5	6622.9182	-0.0002
10	3	8	9	4	5	6626.0911	0.0011
8	6	3	8	5	4	6639.2386	-0.0003
6	1	6	5	0	5	6646.4292	-0.0004
7	6	1	7	5	2	6677.5819	-0.0004
7	6	2	7	5	3	6682.4874	-0.0003
6	6	0	6	5	1	6712.9104	0.0019
6	6	1	6	5	2	6713.7563	-0.0001
4	2	2	3	1	2	6738.3371	-0.0008
10	1	9	10	0	10	6742.0198	0.0003
6	1	5	5	2	4	6760.3082	0.0000
10	2	9	10	1	10	6764.2586	0.0000
3	3	1	2	2	0	6764.9778	-0.0006
3	3	0	2	2	0	6771.1162	0.0003
3	3	1	2	2	1	6807.3857	0.0008
3	3	0	2	2	1	6813.5225	0.0002
13	4	10	13	3	11	6980.6788	-0.0002
5	3	2	5	0	5	6998.3535	0.0001

10	4	7	9	5	4	7032.1716	0.0011
8	3	5	7	4	4	7041.5730	-0.0002
5	2	4	4	1	3	7064.9227	0.0005
12	2	10	12	1	11	7165.1729	-0.0002
12	3	10	12	2	11	7242.8053	-0.0007
7	2	5	6	3	4	7256.2564	-0.0002
6	3	4	5	3	3	7473.8985	0.0003
6	5	2	5	5	1	7478.0096	0.0017
11	1	10	11	0	11	7489.3287	0.0004
11	2	10	11	1	11	7498.7562	0.0011
6	4	3	5	4	2	7506.6100	0.0020
6	4	2	5	4	1	7531.3382	0.0004
12	7	5	12	6	6	7560.6955	-0.0003
13	7	7	13	6	8	7595.2397	-0.0009
7	0	7	6	1	6	7629.4643	-0.0004
7	1	7	6	1	6	7639.0857	0.0000
7	0	7	6	0	6	7652.9744	-0.0015
7	1	7	6	0	6	7662.5969	0.0000
12	7	6	12	6	7	7669.9355	-0.0005
14	4	11	14	3	12	7680.0520	-0.0013
11	7	4	11	6	5	7706.6935	-0.0006
6	3	3	5	3	2	7737.0748	0.0009
11	7	5	11	6	6	7746.7113	0.0000
10	7	3	10	6	4	7801.9829	-0.0004
10	7	4	10	6	5	7814.6116	-0.0001
4	2	2	3	1	3	7864.8501	-0.0003
9	7	2	9	6	3	7865.8222	0.0000
9	7	3	9	6	4	7869.1510	0.0007
6	2	5	5	1	4	7885.6527	0.0002
4	3	2	3	2	1	7905.6358	0.0000
6	2	4	5	2	3	7908.0720	0.0005
8	7	1	8	6	2	7909.3272	0.0013
13	2	11	13	1	12	7941.0606	0.0002

Table S3. Results for $(TFE)_1(CO_2)_3$.

J'	*K*a'	*K*c'	*J"*	*K*a"	*K*c"	ν / MHz	ν_{obs} - ν_{calc} / MHz
2	2	0	1	1	0	2450.0161	-0.0015
2	2	1	1	1	1	2487.1298	-0.0004
2	2	0	1	1	1	2494.1271	0.0008
3	0	3	2	1	2	2592.3451	-0.0003
3	1	3	2	0	2	2844.3191	-0.0001
3	1	2	2	0	2	3108.2585	-0.0002
4	1	3	3	2	2	3268.1743	-0.0012
3	2	2	2	1	1	3321.2022	0.0001
3	2	1	2	1	1	3355.0895	0.0004
3	2	2	2	1	2	3453.5255	-0.0007
3	2	1	2	1	2	3487.4129	-0.0002
4	0	4	3	1	3	3523.4448	0.0000
4	1	4	3	0	3	3693.1510	-0.0004
3	3	1	2	2	0	3798.0803	-0.0003
3	3	0	2	2	0	3798.7848	0.0000
3	3	1	2	2	1	3805.0765	-0.0003
3	3	0	2	2	1	3805.7806	-0.0003
4	1	3	3	0	3	4129.3559	-0.0005
4	2	3	3	1	2	4176.6191	-0.0005
5	1	4	4	2	3	4267.9553	-0.0001
4	2	2	3	1	2	4271.8632	-0.0004
5	0	5	4	1	4	4439.8732	0.0000
4	2	3	3	1	3	4440.5588	-0.0001
5	0	5	4	0	4	4505.2679	0.0028
4	2	2	3	1	3	4535.8023	-0.0007
5	1	5	4	0	4	4542.3594	0.0002
4	3	2	3	2	1	4705.5897	0.0000
4	3	1	3	2	1	4710.4473	0.0006
4	3	2	3	2	2	4739.4764	-0.0001
4	3	1	3	2	2	4744.3341	0.0005
6	2	4	5	3	3	4767.3028	-0.0004
5	2	4	4	1	3	5011.4356	0.0000
5	1	4	4	0	4	5185.0687	-0.0009
5	2	3	4	1	3	5212.3909	-0.0001
6	1	5	5	2	4	5263.4432	0.0002
6	0	6	5	1	5	5342.0624	0.0000
6	1	6	5	1	5	5361.6122	0.0025
6	0	6	5	0	5	5379.1576	0.0011
6	1	6	5	0	5	5398.7038	0.0000
5	2	4	4	1	4	5447.6406	0.0001

5	3	3	4	2	2	5589.5441	0.0000
5	3	2	4	2	2	5608.4019	-0.0002
5	2	3	4	1	4	5648.5955	-0.0004
6	2	4	5	2	3	5655.2198	0.0019
5	3	2	4	2	3	5703.6464	0.0002
7	2	5	6	3	4	5811.1574	-0.0010
6	2	5	5	1	4	5830.6971	-0.0005
5	4	2	4	3	1	6059.0788	-0.0001
5	4	1	4	3	1	6059.6019	0.0002
5	4	2	4	3	2	6063.9363	0.0002
5	4	1	4	3	2	6064.4594	0.0006
6	2	4	5	1	4	6184.1355	-0.0002
7	0	7	6	1	6	6233.8494	0.0001
7	1	6	6	2	5	6243.5588	-0.0003
7	1	7	6	0	6	6263.1788	-0.0001
6	1	5	5	0	5	6271.2096	-0.0004
6	3	4	5	2	3	6443.6726	-0.0005
6	3	3	5	2	3	6497.2872	-0.0003
9	4	5	8	5	4	6590.1282	0.0005
7	2	5	6	2	4	6599.6138	0.0000
7	2	6	6	1	5	6642.9899	-0.0011
6	3	4	5	2	4	6644.6286	0.0000
6	3	3	5	2	4	6698.2441	0.0010
6	2	4	5	1	5	6826.8466	0.0002
8	2	6	7	3	5	6862.1614	-0.0014
6	4	3	5	3	2	6972.8764	-0.0002
6	4	2	5	3	2	6975.4461	0.0002
6	4	3	5	3	3	6991.7348	0.0001
6	4	2	5	3	3	6994.3041	0.0001
8	0	8	7	1	7	7119.1846	-0.0002
8	1	8	7	0	7	7133.6868	0.0001
7	2	5	6	1	5	7192.3444	0.0002
8	1	7	7	2	6	7200.3167	-0.0003
9	3	6	8	4	5	7252.4995	0.0001
7	3	5	6	2	4	7268.2805	-0.0007
7	1	6	6	0	6	7374.9077	0.0027
7	3	4	6	2	4	7391.1087	-0.0009
8	2	7	7	1	6	7459.0134	-0.0005
7	2	6	6	1	6	7515.4976	0.0001
7	3	5	6	2	5	7621.7193	-0.0001
7	3	4	6	2	5	7744.5479	0.0001
7	4	4	6	3	3	7870.6001	0.0002

7	4	3	6	3	3	7879.7626	0.0004
7	4	4	6	3	4	7924.2142	0.0000
7	4	3	6	3	4	7933.3759	-0.0006

Table S4. Results for $(TFE)_1(CO_2)_4$.

J'	*K_a'*	*K_c'*	*J"*	*K_a"*	*K_c"*	ν / MHz	ν_{obs} - ν_{calc} / MHz
3	2	1	2	1	1	2313.4273	-0.0014
3	2	2	2	1	2	2389.2202	0.0013
4	0	4	3	1	3	2466.8314	-0.0021
4	1	4	3	1	3	2493.9034	-0.0022
4	2	3	3	2	2	2577.3801	-0.0001
3	3	1	2	2	1	2599.3173	0.0005
4	1	3	3	0	3	2918.9616	0.0018
4	2	2	3	1	2	2967.4360	0.0003
4	2	3	3	1	3	3090.6740	0.0008
5	0	5	4	1	4	3095.1708	-0.0029
5	2	4	4	2	3	3211.6331	0.0010
4	3	2	3	2	1	3220.0504	0.0004
4	3	1	3	2	1	3227.7490	-0.0003
4	3	2	3	2	2	3258.8202	0.0007
4	3	1	3	2	2	3266.5170	-0.0017
5	1	4	4	1	3	3277.5973	-0.0007
5	2	3	4	2	2	3318.1577	-0.0001
4	4	0	3	3	0	3504.0646	0.0002
4	4	1	3	3	1	3505.0645	-0.0019
5	2	3	4	1	3	3645.5108	-0.0006
5	1	4	4	0	4	3678.8344	0.0002
6	1	6	5	1	5	3719.3863	-0.0001
6	0	6	5	0	5	3726.5760	0.0011
6	1	6	5	0	5	3732.2795	0.0008
5	2	4	4	1	4	3808.3991	-0.0006
5	3	2	4	2	2	3853.9511	-0.0003
6	1	5	5	1	4	3898.6573	0.0009
5	3	3	4	2	3	3929.6178	0.0003
6	3	3	5	3	2	3946.3152	-0.0021
5	3	2	4	2	3	3958.7035	0.0020
6	2	4	5	2	3	3984.7362	0.0012
7	1	6	6	2	4	4051.7148	0.0020
5	4	2	4	3	1	4147.8981	0.0013
5	4	1	4	3	1	4149.0357	0.0003
5	4	2	4	3	2	4155.5955	-0.0005
7	0	7	6	1	6	4326.5180	-0.0003
7	1	7	6	1	6	4328.9226	0.0023
7	0	7	6	0	6	4332.2225	0.0004
7	1	7	6	0	6	4334.6238	-0.0002
6	2	4	5	1	4	4352.6485	0.0001

7	1	6	6	2	5	4407.7603	-0.0019
6	1	5	5	0	5	4455.2444	-0.0003
7	2	6	6	2	5	4462.3770	-0.0012
6	3	3	5	2	3	4482.1110	0.0000
7	1	6	6	1	5	4505.7022	-0.0025
6	2	5	5	1	5	4540.2961	0.0011
7	3	5	6	3	4	4540.6620	-0.0009
7	5	3	6	5	2	4551.1045	-0.0002
7	5	2	6	5	1	4551.8035	-0.0003
7	4	4	6	4	3	4557.4609	0.0018
7	2	6	6	1	5	4560.3181	-0.0026
7	3	4	6	3	3	4630.4762	-0.0011
7	2	5	6	2	4	4638.1674	0.0006
6	3	3	5	2	4	4693.3871	0.0004
6	4	3	5	3	2	4780.5836	-0.0007
6	4	2	5	3	2	4786.0959	-0.0004
6	4	3	5	3	3	4809.6688	0.0005
6	4	2	5	3	3	4815.1808	0.0005
8	0	8	7	1	7	4936.4959	-0.0002
8	1	8	7	1	7	4937.4746	0.0017
8	0	8	7	0	7	4938.8993	0.0012
8	1	8	7	0	7	4939.8750	0.0002
8	1	7	7	2	6	5051.8728	-0.0002
6	5	2	5	4	1	5060.8418	-0.0019
6	5	1	5	4	1	5060.9877	0.0002
8	2	7	7	2	6	5079.6170	0.0003
7	2	5	6	1	5	5092.1590	0.0002
8	1	7	7	1	6	5106.4887	-0.0005
7	3	4	6	2	4	5127.8538	0.0005
8	2	7	7	1	6	5134.2332	0.0004
8	3	6	7	3	5	5178.8350	0.0018
8	5	4	7	5	3	5207.5856	0.0016
8	4	5	7	4	4	5211.9539	0.0000
7	1	6	6	0	6	5234.3738	-0.0007
8	4	4	7	4	3	5245.4362	-0.0004
8	2	6	7	2	5	5274.7234	0.0026
7	2	6	6	1	6	5283.2867	0.0000
8	3	5	7	3	4	5312.6119	-0.0004
7	3	5	6	2	5	5315.2906	0.0003
7	4	4	6	3	3	5391.7268	0.0007
7	4	3	6	3	3	5410.8697	0.0004
7	4	4	6	3	4	5470.5241	0.0000

8	3	6	7	2	5	5499.9052	-0.0021
9	0	9	8	1	8	5545.1507	-0.0024
9	1	9	8	1	8	5545.5424	0.0022
9	0	9	8	0	8	5546.1300	0.0000
9	1	9	8	0	8	5546.5162	-0.0007
9	2	8	8	2	7	5692.8962	-0.0010
7	5	2	6	4	2	5706.1677	0.0002
9	1	8	8	1	7	5707.4818	0.0020
7	5	3	6	4	3	5710.8360	-0.0006
7	5	2	6	4	3	5711.6799	0.0004
9	2	8	8	1	7	5720.6381	-0.0027
8	3	5	7	2	5	5802.3004	0.0014
9	3	7	8	3	6	5810.3148	0.0002
8	2	6	7	1	6	5861.1756	0.0008
9	2	7	8	2	6	5892.7159	-0.0030
8	1	7	7	0	7	6008.6413	-0.0003
8	4	4	7	3	4	6025.8288	0.0003
8	3	6	7	2	6	6031.7457	0.0005
8	4	5	7	3	5	6141.8156	0.0004
10	1	10	9	1	9	6153.3864	0.0024
10	0	10	9	0	9	6153.6201	-0.0006
10	2	9	9	2	8	6303.5303	-0.0005
10	1	9	9	1	8	6310.7489	-0.0006
10	2	9	9	1	8	6316.6938	0.0019
8	5	4	7	4	3	6341.8215	0.0030
8	5	3	7	4	3	6345.3446	-0.0004
10	2	8	9	3	7	6353.1254	-0.0005
8	5	4	7	4	4	6360.9617	0.0002
8	5	3	7	4	4	6364.4879	-0.0003
10	2	8	9	2	7	6495.9079	0.0000
10	4	7	9	4	6	6510.6500	-0.0016
10	5	5	9	5	4	6544.9859	-0.0004
10	3	7	9	3	6	6638.6917	-0.0008
9	2	7	8	1	7	6647.4039	-0.0006
9	3	7	8	2	7	6762.4428	-0.0003
9	1	8	8	0	8	6777.2242	0.0007
9	2	8	8	1	8	6789.4063	-0.0013
9	4	6	8	3	6	6826.6771	-0.0003
11	2	10	10	2	9	6912.5992	0.0004
11	1	10	10	1	9	6915.9565	0.0012
11	2	10	10	1	9	6918.5409	-0.0001
9	5	5	8	4	4	6961.9794	-0.0010

9	5	4	8	4	4	6973.6987	0.0000
9	5	5	8	4	5	7014.6076	0.0012
9	5	4	8	4	5	7026.3242	-0.0004
10	4	7	9	3	6	7051.1005	-0.0027
11	3	9	10	3	8	7054.0857	0.0009
11	2	9	10	2	8	7092.7547	-0.0007
11	3	9	10	2	8	7136.0955	0.0016
11	4	8	10	4	7	7151.2505	0.0014
11	5	7	10	5	6	7181.5306	0.0002
11	5	6	10	5	5	7226.7245	0.0017
10	3	7	9	2	7	7257.3857	-0.0002
9	6	4	8	5	3	7262.3450	0.0002
9	6	3	8	5	3	7262.8943	-0.0004
9	6	4	8	5	4	7265.8705	-0.0010
9	6	3	8	5	4	7266.4233	0.0017
11	3	8	10	3	7	7273.8296	0.0008
10	4	6	9	3	6	7282.6641	0.0016
11	4	7	10	4	6	7310.4149	0.0000
10	2	8	9	1	8	7435.8319	-0.0007
10	3	8	9	2	8	7504.6822	0.0014
10	1	9	9	0	9	7541.8407	-0.0024
10	2	9	9	1	9	7547.3964	-0.0018
10	5	5	9	4	5	7587.8847	0.0013
12	3	10	11	3	9	7668.4534	-0.0029
10	5	6	9	4	6	7675.0411	0.0009
10	6	5	9	5	4	7901.5817	0.0009
10	6	4	9	5	4	7903.7003	-0.0012
10	6	5	9	5	5	7913.2976	-0.0014
11	4	7	10	3	7	7954.3853	0.0004

Table S5. Results for $(TFE)_1(CO_2)_5$.

J'	K_a'	K_c'	*J"*	K_a''	K_c''	ν / MHz	ν_{obs} - ν_{calc} / MHz
4	2	2	3	1	2	2315.79240	0.00020
4	1	3	3	0	3	2318.46580	-0.00220
4	3	1	3	2	1	2378.45600	-0.00100
4	3	2	3	2	2	2394.73020	-0.00070
4	4	0	3	3	0	2461.28010	-0.00090
4	4	1	3	3	1	2462.17660	-0.00120
5	2	3	4	1	3	2885.34610	0.00000
5	1	4	4	0	4	2911.12940	0.00230
5	3	2	4	2	2	2922.25810	0.00020
5	2	4	4	1	4	2933.07480	0.00110
5	3	3	4	2	3	2956.29510	0.00010
5	4	1	4	3	1	3008.03560	0.00000
5	4	2	4	3	2	3013.53890	-0.00010
6	2	4	5	3	2	3188.57120	-0.00180
6	0	6	5	1	5	3214.46440	-0.00070
6	2	4	5	1	4	3467.65620	0.00100
6	3	3	5	2	3	3473.56160	-0.00020
6	1	5	5	0	5	3504.46460	0.00020
6	2	5	5	1	5	3515.15420	0.00010
6	3	4	5	2	4	3524.38780	-0.00040
6	4	2	5	3	2	3550.26400	0.00020
6	4	3	5	3	3	3567.80300	-0.00040
6	5	1	5	4	1	3635.28560	-0.00010
6	5	2	5	4	2	3636.57700	-0.00030
6	5	1	5	4	2	3636.88210	0.00180
7	3	4	6	2	4	4037.02120	0.00030
7	2	5	6	1	5	4060.63050	0.00120
7	4	3	6	3	3	4089.82570	0.00000
7	1	6	6	0	6	4095.51000	0.00340
7	3	5	6	2	5	4098.86330	0.00050
7	2	6	6	1	6	4100.02210	-0.00120
7	4	4	6	3	4	4126.74340	-0.00090
7	5	2	6	4	2	4181.58550	-0.00080
7	5	3	6	4	3	4187.24360	-0.00030
8	2	7	7	1	6	4337.64150	-0.00390
8	4	4	7	3	4	4634.10410	-0.00040
8	2	6	7	1	6	4657.35650	0.00020
8	1	7	7	0	7	4684.58970	0.00130
8	2	7	7	1	7	4686.32590	-0.00080
8	4	5	7	3	5	4691.69690	0.00030

8	5	4	7	4	4	4739.96220	0.00000
8	6	2	7	5	2	4809.19190	-0.00060
8	6	3	7	5	3	4810.56720	-0.00050
8	8	0	7	7	0	4961.12900	0.00030
9	3	6	8	2	6	5205.89920	0.00030
9	2	7	8	1	7	5252.14380	0.00120
9	5	4	8	4	4	5260.04430	-0.00090
9	3	7	8	2	7	5262.32380	0.00270
9	4	6	8	3	6	5263.13300	0.00020
9	2	8	8	1	8	5273.23840	-0.00350
9	5	5	8	4	5	5296.47520	0.00080
9	5	5	8	4	5	5296.47550	0.00110
9	6	3	8	5	3	5355.22990	0.00030
9	6	4	8	5	4	5360.45710	0.00200
9	9	0	8	8	0	5585.94470	-0.00060
10	4	6	9	3	6	5761.60010	0.00040
10	5	5	9	4	5	5798.16200	0.00020
10	3	7	9	2	7	5804.81180	0.00000
11	3	8	10	4	6	5824.26410	0.00050
10	4	7	9	3	7	5840.55000	0.00120
10	2	8	9	1	8	5843.84650	0.00140
10	3	8	9	2	8	5848.19250	-0.00050
10	5	6	9	4	6	5858.41550	-0.00050
10	6	4	9	5	4	5896.69400	0.00050
10	7	3	9	6	3	5982.95460	-0.00070
10	7	4	9	6	4	5984.24940	-0.00040
10	9	1	9	8	1	6135.51000	-0.00010
10	10	0	9	9	0	6210.75010	-0.00070
11	5	6	10	4	6	6346.23570	-0.00070
11	4	7	10	3	7	6348.86520	-0.00040
11	3	8	10	2	8	6403.72680	0.00290
11	4	8	10	3	8	6422.66200	-0.00070
11	5	7	10	4	7	6426.75400	-0.00080
11	6	5	10	5	5	6432.33610	0.00000
11	2	9	10	1	9	6433.43660	-0.00060
11	6	6	10	5	6	6466.03420	0.00120
11	8	3	10	7	3	6608.77320	-0.00040
11	8	4	10	7	4	6609.05190	-0.00100
11	11	0	10	10	0	6835.54350	-0.00120
12	5	7	11	4	7	6909.96310	0.00100
12	4	8	11	3	8	6947.93270	-0.00170
12	6	6	11	5	6	6965.81580	0.00020

12	3	9	11	2	9	6999.03010	-0.00080
12	5	8	11	4	8	7001.50080	0.00330
12	4	9	11	3	9	7007.88640	-0.00030
12	4	9	11	3	9	7007.88660	-0.00010
12	3	10	11	2	10	7022.54250	-0.00280
12	7	5	11	6	5	7070.51520	0.00070
12	7	6	11	6	6	7083.39870	-0.00020
12	8	4	11	7	4	7156.56190	0.00060
12	8	5	11	7	5	7157.70110	-0.00010
12	10	2	11	9	2	7309.76450	0.00000
12	11	1	11	10	1	7385.11860	-0.00050
12	12	0	11	11	0	7460.32930	0.00340
14	1	14	13	0	13	7464.76210	0.00150
13	5	8	12	4	8	7491.28090	-0.00240
13	6	7	12	5	7	7506.01800	0.00040
13	4	9	12	3	9	7550.46870	-0.00230
13	5	9	12	4	9	7581.72880	0.00030
13	6	8	12	5	8	7590.29780	0.00060
13	3	10	12	2	10	7591.02580	-0.00020
13	4	10	12	3	10	7594.82140	0.00180
13	7	6	12	6	6	7605.98910	0.00100
13	7	7	12	6	7	7635.70130	-0.00060
13	8	5	12	7	5	7702.35210	-0.00010
13	8	6	12	7	6	7706.16270	0.00010
13	9	4	12	8	4	7782.48700	0.00030
13	9	5	12	8	5	7782.73820	-0.00180
13	11	2	12	10	2	7934.58580	-0.00140

Table S6. Results for $(TFE)_1(CO_2)_6$.

J'	*K_a'*	*K_c'*	*J"*	*K_a"*	*K_c"*	ν / MHz	ν_{obs} - ν_{calc} / MHz
5	5	1	4	4	0	2565.6702	-0.0010
5	5	0	4	4	1	2567.9467	0.0016
6	4	3	5	3	2	2694.1230	0.0019
8	1	7	7	2	6	2755.6692	0.0017
8	2	7	7	1	6	2758.0522	-0.0040
8	2	6	7	3	5	2885.9867	-0.0034
6	4	2	5	3	3	2889.1284	0.0022
6	5	2	5	4	1	2931.9163	-0.0009
6	5	1	5	4	2	2951.3271	-0.0008
9	1	8	8	2	7	3073.1795	0.0000
9	2	8	8	1	7	3073.8053	-0.0006
6	6	0	5	5	1	3095.7562	-0.0033
9	2	7	8	3	6	3216.1046	0.0022
9	3	7	8	2	6	3231.4255	0.0032
9	3	6	8	4	5	3299.9975	0.0004
7	4	3	6	3	4	3411.9683	-0.0021
9	4	6	8	3	5	3459.1281	-0.0011
7	6	2	6	5	1	3468.1923	-0.0015
10	2	8	9	3	7	3537.1486	0.0042
10	3	8	9	2	7	3541.8053	-0.0005
11	0	11	10	0	10	3559.5263	-0.0014
8	5	4	7	4	3	3565.3311	-0.0017
10	3	7	9	4	6	3661.7738	-0.0030
10	4	7	9	3	6	3727.5980	-0.0014
8	5	3	7	4	4	3812.7247	0.0038
8	6	3	7	5	2	3828.7547	-0.0008
11	2	9	10	3	8	3855.0123	0.0040
11	3	9	10	2	8	3856.3291	0.0013
8	6	2	7	5	3	3858.8876	0.0019
12	0	12	11	0	11	3876.4087	-0.0011
11	3	8	10	4	7	3996.6032	-0.0044
11	4	8	10	3	7	4020.1385	0.0039
10	5	6	9	4	5	4042.2850	-0.0031
9	6	4	8	5	3	4157.7435	0.0017
12	2	10	11	3	9	4171.9387	0.0000
13	0	13	12	0	12	4193.2888	-0.0014
9	6	3	8	5	4	4268.3412	0.0020
12	3	9	11	4	8	4319.3102	0.0011
12	4	9	11	3	8	4326.9043	-0.0001
13	1	12	12	2	11	4340.6638	0.0023

9	7	3	8	6	2	4369.4740	0.0019
9	7	2	8	6	3	4378.6367	-0.0045
10	6	5	9	5	4	4436.8245	-0.0019
14	0	14	13	0	13	4510.1666	-0.0014
13	4	10	12	3	9	4639.7989	-0.0005
10	7	4	9	6	3	4724.3440	0.0033
10	7	3	9	6	4	4765.1309	0.0031
13	4	9	12	5	8	4777.7050	-0.0007
13	5	9	12	4	8	4810.7110	0.0008
15	0	15	14	0	14	4827.0418	-0.0009
10	8	3	9	7	2	4902.3421	0.0009
14	3	11	13	4	10	4954.3461	0.0002
14	4	11	13	3	10	4954.9927	-0.0009
15	1	14	14	2	13	4974.3525	0.0023
11	7	5	10	6	4	5043.7024	0.0015
14	4	10	13	5	9	5102.1666	-0.0020
14	5	10	13	4	9	5113.3358	-0.0005
11	8	4	10	7	3	5269.5955	-0.0017
11	8	3	10	7	4	5282.6773	0.0000
12	7	6	11	6	5	5308.7481	0.0018
14	6	9	13	5	8	5327.2306	0.0004
15	5	11	14	4	10	5424.2501	0.0000
11	9	3	10	8	2	5432.4259	0.0004
11	9	2	10	8	2	5432.5551	0.0016
11	9	2	10	8	3	5433.0706	-0.0035
12	8	5	11	7	4	5618.9273	0.0012
12	8	4	11	7	5	5670.1248	0.0018
12	9	4	11	8	3	5804.5177	-0.0038
12	9	3	11	8	4	5808.3297	-0.0030
13	8	6	12	7	5	5929.2732	0.0039
13	9	5	12	8	4	6168.7080	0.0007
13	10	4	12	9	3	6335.5329	-0.0015
13	10	3	12	9	4	6336.5676	-0.0027
14	9	5	13	8	6	6573.9719	-0.0023
14	10	4	13	9	5	6711.0225	0.0020
15	11	5	14	10	4	7237.9045	0.0027
15	11	4	14	10	5	7239.3793	-0.0014

Table S7. Results for $(TFE)_2(CO_2)_1$.

J'	*K_a'*	*K_c'*	*J"*	*K_a"*	*K_c"*	ν / MHz	ν_{obs} - ν_{calc} / MHz
3	1	3	2	1	2	2517.2631	0.0011
3	0	3	2	0	2	2602.8944	0.0003
3	2	2	2	2	1	2773.3770	-0.0006
3	2	1	2	2	0	2943.8702	0.0006
3	1	2	2	1	1	2964.8091	0.0000
8	3	6	8	1	7	3155.1308	-0.0003
7	1	6	7	1	7	3171.2017	0.0007
7	2	6	7	0	7	3211.9280	-0.0006
4	1	4	3	1	3	3317.7731	0.0002
4	0	4	3	0	3	3364.7242	0.0006
4	2	3	3	2	2	3658.5514	-0.0001
4	3	2	3	3	1	3772.4710	0.0001
4	3	1	3	3	0	3830.8618	-0.0002
4	1	3	3	1	2	3870.1740	-0.0006
4	2	2	3	2	1	3989.8024	0.0000
5	1	5	4	1	4	4103.4795	-0.0002
5	0	5	4	0	4	4123.3143	-0.0004
9	1	8	9	1	9	4211.4849	-0.0006
9	2	8	9	0	9	4216.6959	0.0008
5	2	4	4	2	3	4513.9008	-0.0006
5	1	4	4	1	3	4695.5144	0.0002
5	3	3	4	3	2	4711.0081	0.0000
5	4	2	4	4	1	4730.5701	0.0010
5	4	1	4	4	0	4744.7164	-0.0002
6	1	6	5	1	5	4880.9644	0.0000
5	3	2	4	3	1	4882.1637	0.0000
6	0	6	5	0	5	4888.2161	0.0002
5	2	3	4	2	2	4998.7092	0.0002
6	2	5	5	2	4	5339.6329	0.0003
6	1	5	5	1	4	5456.6777	0.0001
6	3	4	5	3	3	5626.2801	0.0000
7	1	7	6	1	6	5654.7345	0.0001
7	0	7	6	0	6	5657.1732	0.0000
6	5	2	5	5	1	5674.5968	0.0013
6	5	1	5	5	0	5677.4382	-0.0017
6	4	3	5	4	2	5696.8901	0.0000
6	4	2	5	4	1	5754.5771	0.0000
6	2	4	5	2	3	5940.3768	0.0003
6	3	3	5	3	2	5963.8961	0.0000
7	2	6	6	2	5	6141.3300	0.0000

7	1	6	6	1	5	6200.1388	-0.0002
8	1	8	7	1	7	6427.0649	-0.0002
8	0	8	7	0	7	6427.8428	0.0001
7	3	5	6	3	4	6509.8363	-0.0003
7	6	1	6	6	0	6615.9406	-0.0006
7	5	3	6	5	2	6648.8667	-0.0002
7	4	4	6	4	3	6656.2494	-0.0005
7	5	2	6	5	1	6663.5292	-0.0009
7	2	5	6	2	4	6794.3818	0.0004
7	4	3	6	4	2	6816.6071	-0.0004
8	2	7	7	2	6	6927.2061	0.0000
8	1	7	7	1	6	6952.1680	-0.0011
7	3	4	6	3	3	7018.6202	0.0002
9	1	9	8	1	8	7198.8963	0.0001
9	0	9	8	0	8	7199.1355	0.0005
8	3	6	7	3	5	7359.1744	-0.0001
8	2	6	7	2	5	7565.0005	0.0000
8	6	3	7	6	2	7589.5629	0.0011
8	6	2	7	6	1	7592.7272	-0.0006
8	4	5	7	4	4	7595.6097	-0.0003
8	5	4	7	5	3	7628.5261	0.0025
8	5	3	7	5	2	7681.1906	0.0001
9	2	8	8	2	7	7704.5377	-0.0003
9	1	8	8	1	7	7714.0768	0.0000
8	4	4	7	4	3	7922.2810	-0.0006

Table S8. Results for $(TFE)_2(CO_2)_2$.

J'	*K_a'*	*K_c'*	*J''*	*K_a''*	*K_c''*	ν / MHz	ν_{obs} - ν_{calc} / MHz
4	1	4	3	1	3	2583.8114	0.0022
4	0	4	3	0	3	2610.9411	-0.0007
4	2	3	3	2	2	2623.8313	0.0021
4	3	2	3	3	1	2627.6641	0.0027
4	3	1	3	3	0	2628.0713	0.0011
4	2	2	3	2	1	2637.8356	-0.0015
4	1	3	3	1	2	2660.2858	-0.0011
4	1	3	3	0	3	2915.1664	-0.0029
5	1	5	4	1	4	3227.4571	-0.0003
5	0	5	4	0	4	3254.3988	0.0011
5	2	4	4	2	3	3277.9608	0.0008
5	3	3	4	3	2	3285.4787	0.0013
5	3	2	4	3	1	3286.8984	0.0011
5	2	3	4	2	2	3304.7108	0.0008
5	1	4	4	1	3	3322.1486	-0.0003
4	3	1	3	2	1	3576.6832	0.0007
4	3	2	3	2	2	3583.3919	0.0005
4	3	1	3	2	2	3583.8668	-0.0017
6	1	6	5	1	5	3869.8920	0.0000
6	0	6	5	0	5	3893.7986	-0.0004
6	2	5	5	2	4	3930.8919	0.0003
6	5	1	5	5	0	3941.0284	-0.0008
6	4	3	5	4	2	3942.2817	-0.0033
6	4	2	5	4	1	3942.3799	-0.0009
6	3	4	5	3	3	3943.5719	0.0006
6	3	3	5	3	2	3947.3027	-0.0005
6	1	6	5	0	5	3955.4406	-0.0034
6	2	4	5	2	3	3974.3690	0.0008
6	1	5	5	1	4	3981.4988	0.0013
5	3	3	4	2	2	4223.8485	0.0030
5	3	2	4	2	2	4225.7447	0.0021
5	3	3	4	2	3	4245.0381	-0.0013
6	1	5	5	0	5	4353.4753	-0.0008
6	2	4	5	1	4	4457.8654	0.0005
7	0	7	6	1	6	4468.7559	-0.0003
7	1	7	6	1	6	4511.1390	-0.0007
7	0	7	6	0	6	4530.4024	0.0012
7	2	6	6	2	5	4582.4172	-0.0003
7	6	1	6	6	0	4597.6664	-0.0008
7	4	4	6	4	3	4600.6737	0.0021

7	4	3	6	4	2	4600.9881	-0.0004
7	3	5	6	3	4	4601.7286	-0.0003
7	3	4	6	3	3	4609.9220	0.0006
7	1	6	6	1	5	4637.5115	0.0014
7	2	5	6	2	4	4645.2563	0.0004
6	3	4	5	2	3	4862.7091	0.0023
6	3	3	5	2	3	4868.3373	0.0014
6	3	3	5	2	4	4916.2800	0.0000
7	1	6	6	0	6	5097.1851	-0.0020
8	1	8	7	1	7	5151.3071	-0.0022
8	0	8	7	0	7	5165.6496	0.0014
8	2	7	7	2	6	5232.3668	0.0005
8	7	1	7	7	0	5254.3076	-0.0009
8	6	2	7	6	1	5255.2554	0.0012
8	4	4	7	4	3	5260.4445	0.0020
6	4	3	5	3	2	5273.6490	0.0025
6	4	2	5	3	2	5273.7671	0.0008
8	3	5	7	3	4	5275.4076	-0.0001
6	4	3	5	3	3	5275.5440	0.0003
8	1	7	7	1	6	5289.3375	0.0007
8	2	6	7	2	5	5315.5778	0.0007
7	3	4	6	2	4	5503.8874	-0.0016
8	2	7	7	1	6	5562.2183	-0.0021
7	3	5	6	2	5	5581.4855	-0.0026
9	0	9	8	1	8	5772.5567	-0.0001
9	1	9	8	1	8	5790.5611	0.0001
9	0	9	8	0	8	5800.6025	0.0009
9	1	9	8	0	8	5818.6021	-0.0035
8	1	7	7	0	7	5856.1227	0.0000
9	2	8	8	2	7	5880.6165	0.0000
9	8	1	8	8	0	5910.9413	-0.0024
9	6	3	8	6	2	5913.1541	-0.0002
9	3	7	8	3	6	5917.0057	-0.0015
9	4	6	8	4	5	5918.9794	-0.0007
9	4	5	8	4	4	5921.0162	0.0000
7	4	3	6	3	3	5927.4513	-0.0002
7	4	4	6	3	4	5932.6412	-0.0027
9	1	8	8	1	7	5936.3203	0.0000
9	3	6	8	3	5	5944.2203	0.0030
9	2	7	8	2	6	5983.8404	-0.0011
8	2	7	7	1	7	6086.6246	0.0016
8	3	5	7	2	5	6134.0387	-0.0022

8	3	5	7	2	6	6288.2985	-0.0014
10	1	9	9	2	8	6361.1419	-0.0020
10	1	10	9	1	9	6429.0743	0.0001
10	0	10	9	0	9	6435.7910	0.0010
9	2	7	8	1	7	6494.1944	-0.0006
10	2	9	9	2	8	6527.1118	-0.0004
10	8	2	9	8	1	6568.3614	0.0006
10	7	3	9	7	2	6569.5425	-0.0003
10	3	8	9	3	7	6573.4263	-0.0020
10	5	6	9	5	5	6574.5146	0.0001
10	5	5	9	5	4	6574.7004	0.0010
10	4	7	9	4	6	6578.7744	-0.0004
10	4	6	9	4	5	6583.0845	0.0004
10	3	7	9	3	6	6616.2692	0.0000
10	2	8	9	2	7	6648.9884	-0.0003
9	3	6	8	2	6	6762.6841	0.0029
9	2	8	8	1	8	6815.9319	0.0017
11	1	10	10	2	9	7050.0152	0.0002
11	0	11	10	1	10	7060.0767	0.0025
11	1	11	10	1	10	7067.0225	0.0015
11	0	11	10	0	10	7071.3636	0.0012
11	1	10	10	1	9	7215.9835	0.0001
11	9	2	10	9	1	7224.9239	0.0035
11	8	3	10	8	2	7225.9822	-0.0002
11	7	4	10	7	3	7227.5638	0.0002
11	3	9	10	3	8	7228.5822	-0.0007
11	5	6	10	5	5	7234.5835	-0.0004
11	4	8	10	4	7	7238.7896	-0.0028
11	4	7	10	4	6	7247.1325	0.0005
9	4	6	8	3	6	7249.8178	-0.0021
11	3	8	10	3	7	7290.6845	0.0012
11	2	9	10	2	8	7310.2231	-0.0003
12	0	12	11	0	11	7707.2829	-0.0015
8	7	1	7	6	1	7732.9191	0.0000
12	2	11	11	2	10	7814.9861	-0.0005
12	1	11	11	1	10	7850.6165	-0.0017
12	3	10	11	3	9	7882.1824	0.0006
12	9	3	11	9	2	7882.4419	-0.0020
12	8	4	11	8	3	7883.8307	0.0004
12	5	8	11	5	7	7894.2860	0.0012
12	5	7	11	5	6	7895.3116	0.0009
12	4	9	11	4	8	7898.7887	0.0022

12	4	8	11	4	7	7913.7283	0.0006
12	3	9	11	3	8	7965.8524	-0.0014

IV. Computational Results for all Observed Clusters. Computed principal axis coordinates, rotational constants, and dipole moment components for the favored structure (closest agreement with experimental data) of each observed cluster. All reported results are at the B3LYP-D3BJ/6-311++G(2d,2p) level without BSSE or ZPE corrections.

a. $(TFE)_1(CO_2)_1$ (previously studied in Ref. 10); Figure 4(a) of main article.

E(RB3LYP) = -565.077477660 E_h (2nd lowest energy structure, ΔE = 9 cm^{-1})

Center Number	Atomic Number	Coordinates (Angstroms) X	Y	Z
1	6	1.962014	0.079820	-0.000031
2	6	0.656004	0.271629	-0.000464
3	1	0.189733	1.240813	-0.000744
4	9	2.834503	1.073338	0.000115
5	9	2.555919	-1.095554	0.000380
6	9	-0.182880	-0.783096	-0.000520
7	6	-2.964098	0.220195	0.000133
8	8	-2.434665	1.254588	0.000135
9	8	-3.503048	-0.805109	0.000213

```
Rotational constants (MHZ):
  5320.9289464    696.7832590    616.1036285
Nuclear quadrupole coupling constants [Chi] (MHz):
Dipole moment (Debye):
    -0.3961970      1.1846728     -0.0004240  Tot=      1.2491685
```

b. $(TFE)_1(CO_2)_1$ (2nd isomer); Figure 4(b) of main article.

E(RB3LYP) = -565.077272229 E_h (3rd lowest energy structure, ΔE = 54 cm^{-1})

Center Number	Atomic Number	Coordinates (Angstroms) X	Y	Z
1	6	-1.289449	-0.831869	0.000273
2	1	-0.452633	-1.508086	0.000324
3	6	-1.161431	0.482052	0.000220
4	9	0.016883	1.092244	0.000042
5	9	-2.155278	1.345647	-0.000202
6	9	-2.510597	-1.392764	-0.000051
7	6	2.685336	-0.318960	-0.000030
8	8	3.308159	0.657628	0.000076
9	8	2.066433	-1.302241	-0.000191

Rotational constants (MHZ):
3544.8473793 880.0958903 705.0498611
Nuclear quadrupole coupling constants [Chi] (MHz):
Dipole moment (Debye):
1.2034100 -0.6751254 0.0004107 Tot= 1.3798514

c. $(TFE)_1(CO_2)_2$; Figure 4(c) of main article.

E(RB3LYP) = -753.734903086 E_h (lowest energy structure)

Center Number	Atomic Number	Coordinates (Angstroms) X	Y	Z
1	6	2.059055	-0.412950	-0.062526
2	6	1.062865	-1.135644	-0.537101
3	1	0.953167	-2.193096	-0.377276
4	9	3.047948	-0.919964	0.650083
5	9	2.190687	0.888201	-0.233108
6	9	0.084816	-0.541013	-1.251361
7	6	-2.419320	-1.202112	0.138741
8	8	-1.852835	-2.147752	0.501329
9	8	-2.993820	-0.262069	-0.223454
10	6	-0.750892	1.922917	0.390787
11	8	-0.603416	1.154258	1.249547
12	8	-0.896833	2.695118	-0.459995

Rotational constants (MHZ):
1230.2742632 717.0293640 526.3258955
Nuclear quadrupole coupling constants [Chi] (MHz):
Dipole moment (Debye):
0.1525718 -1.3458572 0.2421154 Tot= 1.3759468

d. $(TFE)_1(CO_2)_3$; Figure 4(d) of main article.

E(RB3LYP) = -942.392465980 E_h (lowest energy structure)

Center Number	Atomic Number	Coordinates (Angstroms) X	Y	Z
1	6	-2.455530	-1.053224	0.834286
2	8	-1.996064	-1.759777	1.631822
3	8	-2.922162	-0.346670	0.042157
4	6	-1.117080	1.909303	-1.061405
5	8	-0.478614	1.085989	-1.574775
6	8	-1.749088	2.739978	-0.559743
7	6	0.598374	-1.662867	-1.577722
8	8	-0.387503	-2.067265	-1.116295
9	8	1.589292	-1.273769	-2.034969
10	6	1.988910	0.311606	0.879623
11	6	0.859744	-0.085916	1.437194
12	1	0.709872	-1.054256	1.880406
13	9	3.065474	-0.449178	0.816530
14	9	2.183118	1.491929	0.332248
15	9	-0.202508	0.745381	1.468898

Rotational constants (MHZ):
671.5154534 489.4062788 440.2122119
Nuclear quadrupole coupling constants [Chi] (MHz):
Dipole moment (Debye):
0.1659513 -1.1067432 0.5072657 Tot= 1.2287143

e. $(TFE)_1(CO_2)_4$; Figure 4(e) of main article.

E(RB3LYP) = -1131.05059998 E_h (2nd lowest energy structure, ΔE = 22 cm^{-1})

Center Number	Atomic Number	Coordinates (Angstroms) X	Y	Z
1	6	-0.363522	-2.025036	0.417039
2	6	0.952637	-2.079627	0.359924
3	1	-0.939972	-2.210695	1.305291
4	9	-1.070247	-1.694830	-0.685233
5	9	1.668640	-1.829492	-0.721357
6	9	1.711111	-2.397209	1.390598
7	6	-0.302035	1.951085	1.600562
8	8	0.209008	2.950127	1.313260
9	8	-0.823325	0.957367	1.899788
10	6	2.838485	1.018595	-0.028283
11	8	2.368077	0.668559	0.974335
12	8	3.317491	1.365710	-1.024220
13	6	-3.204201	0.040162	0.350220
14	8	-3.422152	-0.826886	1.088362
15	8	-2.995324	0.910765	-0.388175
16	6	-0.468358	0.712825	-2.163006
17	8	-1.048875	0.385757	-3.109244
18	8	0.121525	1.047947	-1.219827

Rotational constants (MHZ):
459.8739132 346.2616164 310.8468698
Nuclear quadrupole coupling constants [Chi] (MHz):
Dipole moment (Debye):
-0.5106382 -0.3683221 1.1397759 Tot= 1.3021143

f. $(TFE)_1(CO_2)_5$; Figure 4(f) of main article.

E(RB3LYP) = -1319.70955148 E_h (lowest energy structure)

Center Number	Atomic Number	Coordinates (Angstroms) X	Y	Z
1	6	-1.969133	-0.831298	1.334730
2	6	-2.550458	0.020739	0.513070
3	1	-1.825278	-0.650557	2.384249
4	9	-1.489597	-2.001609	0.861803
5	9	-2.701922	-0.175537	-0.786466
6	9	-3.051612	1.176419	0.896152
7	6	0.737183	1.830925	2.083657
8	8	1.753283	1.978369	1.544297
9	8	-0.274724	1.692152	2.632632
10	6	-0.135798	-1.753487	-1.779520
11	8	-0.597824	-2.673711	-2.305240
12	8	0.331122	-0.825024	-1.257185
13	6	-0.521229	1.957845	-1.789229
14	8	-0.535708	2.174030	-0.648654
15	8	-0.521309	1.745912	-2.928173
16	6	2.894793	0.661834	-0.873465
17	8	3.285989	-0.206480	-0.211228
18	8	2.512443	1.525335	-1.546401
19	6	1.428464	-2.032462	1.377650
20	8	1.204126	-1.155727	2.104781
21	8	1.648003	-2.915760	0.660652

Rotational constants (MHZ):
316.9138844 283.9779294 270.1475311

Nuclear quadrupole coupling constants [Chi] (MHz):

Dipole moment (Debye):
-0.0295894 0.2935514 1.2610340 Tot= 1.2950887

g. $(TFE)_1(CO_2)_6$; Figure 4(g) of main article.

E(RB3LYP) = -1508.36643628 E_h (4th lowest energy structure, ΔE = 329 cm^{-1})

Center Number	Atomic Number	Coordinates (Angstroms) X	Y	Z
1	6	2.737911	2.146631	0.318091
2	6	2.328889	1.923973	-0.915740
3	1	2.355766	2.936863	0.938988
4	9	3.661440	1.341838	0.878103
5	9	2.752651	0.943092	-1.690113
6	9	1.416439	2.663808	-1.521795
7	6	-0.422972	-0.036661	-1.734938
8	8	-0.102416	0.130144	-0.629523
9	8	-0.734038	-0.201036	-2.835975
10	6	-1.870559	-1.851570	0.882972
11	8	-2.638180	-1.560996	0.063533
12	8	-1.106476	-2.153634	1.701113
13	6	-1.155023	1.882222	1.600149
14	8	-1.907457	1.039984	1.868462
15	8	-0.417251	2.736136	1.337831
16	6	-3.930574	1.095165	-0.591609
17	8	-2.934865	1.492694	-1.039952
18	8	-4.928964	0.712768	-0.147235
19	6	1.596306	-0.728317	1.739357
20	8	2.055119	-1.571273	1.089482
21	8	1.142477	0.115058	2.393784
22	6	0.971030	-3.679078	-0.745958
23	8	1.786001	-4.420219	-0.391175
24	8	0.145370	-2.947128	-1.111842

Rotational constants (MHZ):
251.1852564 209.1434938 163.0854841
Nuclear quadrupole coupling constants [Chi] (MHz):
Dipole moment (Debye):
-0.5247077 0.8705888 0.5850294 Tot= 1.1728182

h. $(TFE)_2(CO_2)_1$; Figure 4(h) of main article.

E(RB3LYP) = -941.506776866 E_h (lowest energy structure)

Center Number	Atomic Number	Coordinates (Angstroms) X	Y	Z
1	6	1.203323	-1.731824	0.058582
2	6	-0.116102	-1.725632	0.061135
3	1	-0.723011	-2.112226	-0.737942
4	9	1.921535	-2.235611	-0.928343
5	9	1.961674	-1.243966	1.020546
6	9	-0.784856	-1.189696	1.102149
7	6	-3.229903	-0.000099	-0.000126
8	8	-3.235894	-0.908480	-0.721561
9	8	-3.236758	0.908266	0.721347
10	6	-0.117147	1.724839	-0.061223
11	1	-0.724624	2.110922	0.737662
12	6	1.202274	1.732296	-0.058526
13	9	1.919904	2.237434	0.928129
14	9	1.961172	1.244734	-1.020196
15	9	-0.785270	1.187619	-1.101991

Rotational constants (MHZ):
818.0709552 536.2165325 392.5206213
Nuclear quadrupole coupling constants [Chi] (MHz):
Dipole moment (Debye):
-1.0596371 -0.0002795 -0.0005511 Tot= 1.0596373

i. $(TFE)_2(CO_2)_2$; Figure 4(i) of main article.

E(RB3LYP) = -1130.16430067 E_h (lowest energy structure)

Center Number	Atomic Number	Coordinates (Angstroms) X	Y	Z
1	6	-1.674629	1.436907	0.956291
2	6	-0.497174	1.753419	0.452523
3	1	-0.333555	2.541445	-0.260270
4	9	-2.787557	2.075082	0.647239
5	9	-1.887805	0.459766	1.814859
6	9	0.598371	1.052542	0.815376
7	6	2.396121	1.020018	-1.473609
8	8	2.023020	2.095460	-1.692742
9	8	2.780123	-0.053824	-1.258247
10	6	-0.385754	-1.266757	-1.198166
11	1	0.490258	-1.809828	-0.892880
12	6	-1.604747	-1.530311	-0.767840
13	9	-1.879524	-2.512965	0.069685
14	9	-2.687360	-0.857035	-1.108155
15	9	-0.181945	-0.239946	-2.050319
16	6	2.542507	-1.132007	1.569435
17	8	1.903391	-2.019037	1.178409
18	8	3.184278	-0.252925	1.967257

Rotational constants (MHZ):
525.8657704 339.5514693 320.3607365
Nuclear quadrupole coupling constants [Chi] (MHz):
Dipole moment (Debye):
0.8652563 0.3474026 -0.3442966 Tot= 0.9939301

V. *IvI* and *RvI* Plots with Overlays of All Assigned Transitions and Analysis of Observed Ratio Ranges.

Figure S2. (a) Plot of *Intensity in 2% CO_2 Scan* vs. *Intensity in 1% CO_2 Scan* (*IvI* plot) for all transitions with S/N ≳ 2, showing overlays for all assigned clusters. (b) *RvI* plot showing ratio (*Intensity in 1% CO_2 Scan*)/(*Intensity in 2% CO_2 Scan*) *vs. Intensity in 1% CO_2 Scan*. Insets zoom out to show ~6x the intensity range.

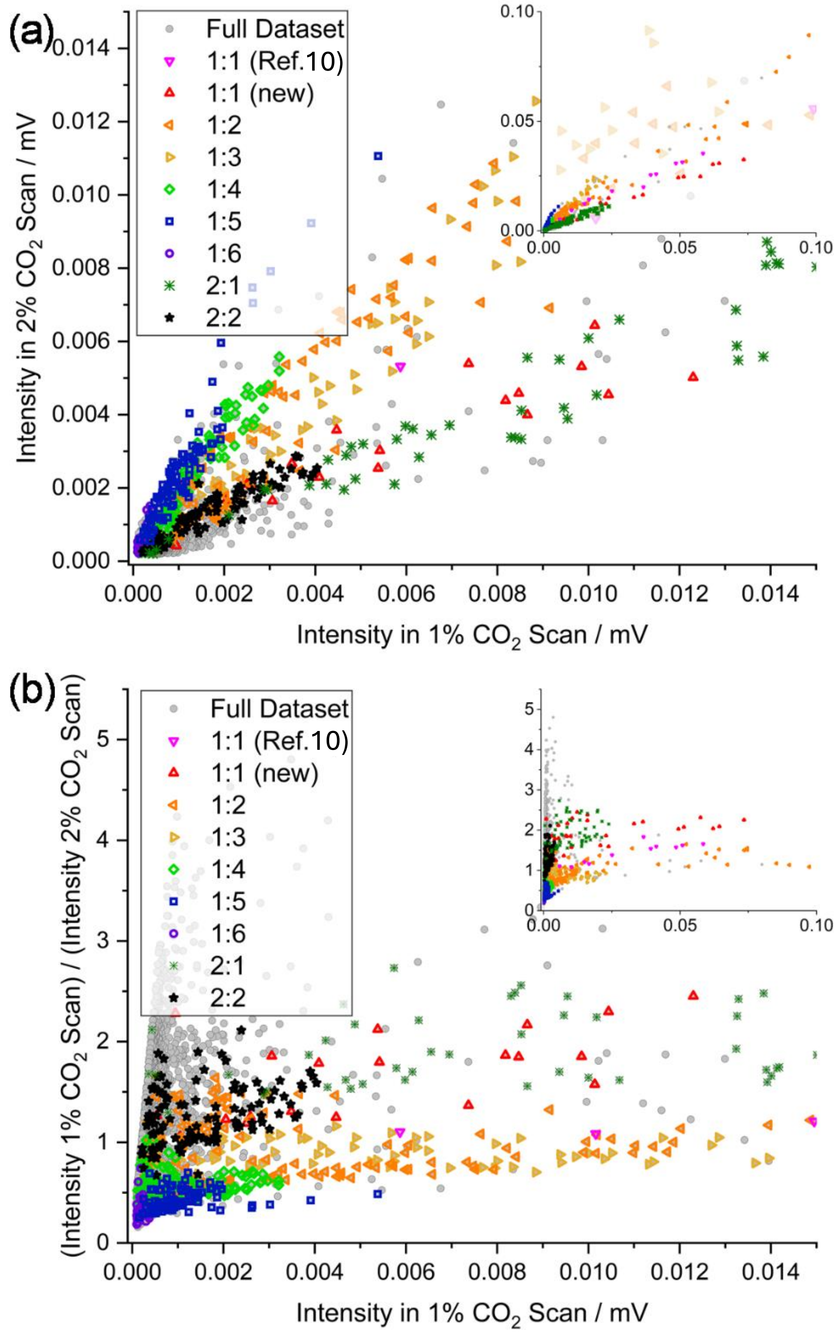

Figure S3. Plot of Weighted R vs. # of CO_2 in cluster for $(TFE)_1(CO_2)_n$ clusters. Weighted $R = \frac{\sum R_i I_i}{\sum I_i}$, where summations are over all fitted transitions for each cluster, R_i is intensity ratio of 1% CO_2 scan / 2% CO_2 scan, and I_i is intensity in the 1% CO_2 scan.

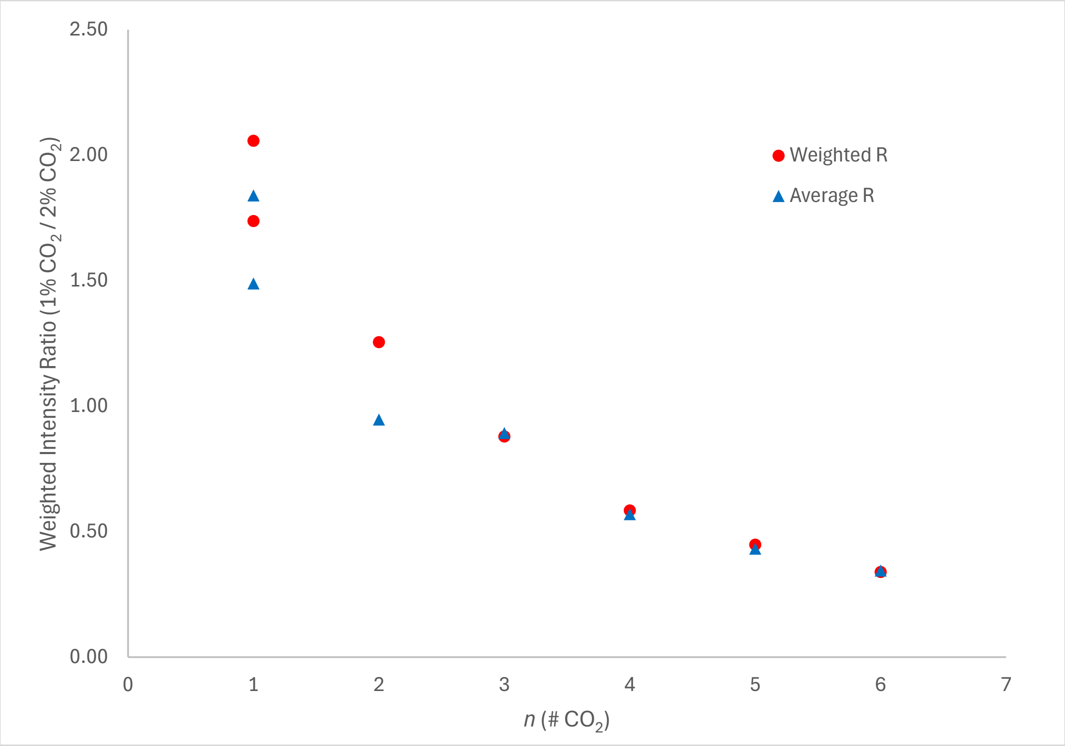

Table S10. Statistical details related to intensity-ratio analysis for assigned cluster species.

	**$(TFE)_1$-$(CO_2)_1$** [a]	**$(TFE)_1$-$(CO_2)_1$**	**$(TFE)_1$-$(CO_2)_2$**	**$(TFE)_1$-$(CO_2)_3$**	**$(TFE)_1$-$(CO_2)_4$**	**$(TFE)_1$-$(CO_2)_5$**	**$(TFE)_1$-$(CO_2)_6$**	**$(TFE)_2$-$(CO_2)_1$**	**$(TFE)_2$-$(CO_2)_2$**
m:n [b]	1:1	1:1	1:2	1:3	1:4	1:5	1:6	2:1	2:2
Weighted R [c]	1.74	2.06	1.25	0.88	0.58	0.45	0.34	2.00	1.34
Average R [d]	1.49	1.84	0.95	0.89	0.57	0.43*	0.35*	1.95	1.27
Std. Dev. R [d]	0.30	0.40	0.34	0.12	0.13	0.10	0.13	0.34	0.29
R range (extracted) [e]	1.25-2.40	1.25-2.75	1.25-2.40	0.60-1.35	0.15-1.85	0.29-0.56	0.15-0.50	0.85-3.50	1.00-3.50
R range (assigned) [f]	1.09-2.09	1.06-2.45	0.44-1.66	0.58-1.16	0.29-1.02	0.25-0.70	0.19-0.74	1.30-2.73	0.67-2.11

[a] Experimental results reported in Ref. 10 of main article.

[b] Ratio of # of TFE molecules (m) to # of CO_2 molecules (n) in each assigned cluster.

[c] Weighted $R = \frac{\sum R_i I_i}{\sum I_i}$, where summations are over all fitted transitions for each cluster, R_i is intensity ratio of 1% CO_2 scan / 2% CO_2 scan, and I_i is intensity in the 1% CO_2 scan. Entries marked * were analyzed using the 2% CO_2 scan as parent.

[d] Average and standard deviation of intensity ratio of all fitted transitions for each cluster.

[e] Range of ratio values extracted for analysis that led to assignment of each cluster. Figures 2 and 3 of the main article show *RvI* plots with assigned transitions overlayed and spectra of extracted transitions within the listed ratio range, respectively.

[f] Range of ratio values of fitted transitions for each cluster.

VI. Comparison of *RvI* Plots Generated with 1% and 2% CO_2 Parent Spectrum.

Figure S4. Comparison of *RvI* plots for 2% CO_2 parent spectrum and 1% CO_2 parent spectrum, highlighting the n=5 cluster transitions (blue squares) on each plot. Also clearly visible are transitions for $(TFE)_2(CO_2)_1$ (green asterisks). Transitions for TFE-only and n=1-4 clusters were removed from the dataset prior to generating both plots.

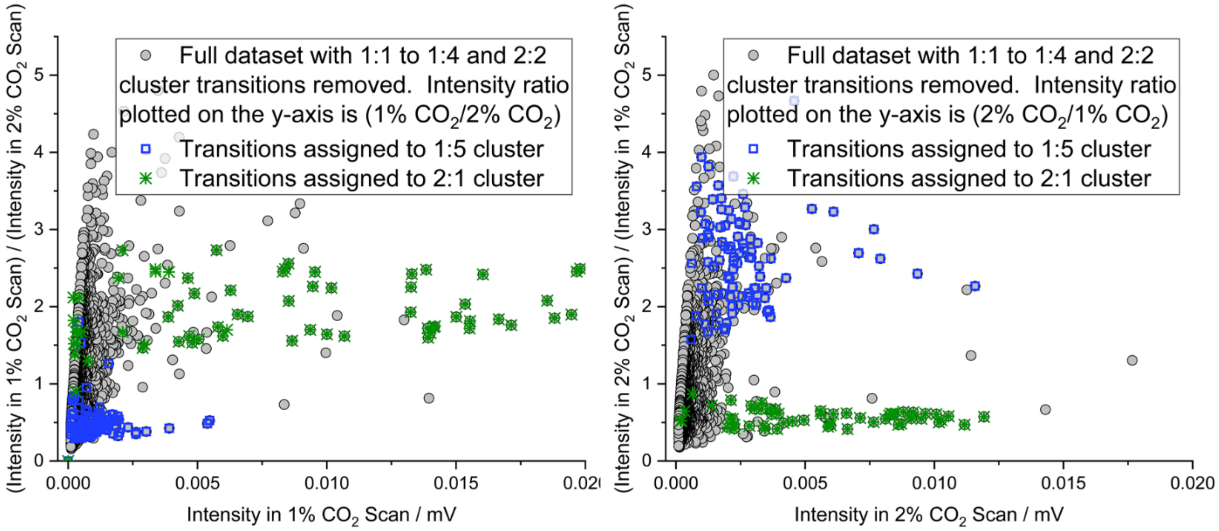

Supplement: Supplementary file 1 [file jp5c05251_si_001.pdf]
